# Nanomicelle protects the immune activation effects of Paclitaxel and sensitizes tumors to anti-PD-1 Immunotherapy

**DOI:** 10.7150/thno.45391

**Published:** 2020-07-09

**Authors:** Qianmei Yang, Gang Shi, Xiaolei Chen, Yi Lin, Lin Cheng, Qingyuan Jiang, Xi Yan, Ming Jiang, Yiming Li, Hantao Zhang, Huiling Wang, Yuan Wang, Qingnan Wang, Yujing Zhang, Yi Liu, Xiaolan Su, Lei Dai, Minghai Tang, Jia Li, Lan Zhang, Zhiyong Qian, Dechao Yu, Hongxin Deng

**Affiliations:** 1 State Key Laboratory of Biotherapy and Cancer Center, West China Hospital, Sichuan University, Chengdu, 610041, P.R. China.; 2 School of Pharmaceutical Science & Yunnan Key Laboratory of Pharmacology for Natural Products, Kunming Medical University, Kunming, Yunnan, 650500, P.R. China.; 3 Department of Obstetrics, Sichuan Provincial Hospital for Women and Children, Chengdu, Sichuan, P.R. China.; 4 Department of medical Oncology, Cancer Center, West China Hospital, Sichuan University, Chengdu, Sichuan 610041, China.; 5 Innovent Biologics, Inc., Suzhou, Jiangsu, P.R. China.; 6 Guangdong Zhongsheng Pharmaceutical Co., Ltd. China.

**Keywords:** Paclitaxel (PTX), Nanomicelle, Immunogenic cell death (ICD), anti-PD-1 immunotherapy, Combination immunotherapy

## Abstract

Paclitaxel (PTX) has shown pleiotropic immunologic effects on the tumor microenvironment, and nanomicelle has emerged as a promising strategy for PTX delivery. However, the detailed mechanisms remain to be fully elucidated. Meanwhile, immunogenic cell death (ICD) is an effective approach to activate the immune system. This study investigated the ICD effect of PTX and how nanomicelle affected the immune-activation ability of PTX.

**Methods:** The ICD effects of PTX were identified via the expression of ICD markers and cell vaccine experiment. Tumor size and overall survival in multiple animal models with treatment were monitored to evaluate the antitumor effects. The mechanisms of PTX-induced ICD and antitumor immunity were determined by detecting gene expression related to ER stress and analyzing immune cell profile in tumor after treatment.

**Results:** We revealed the immune-regulation mechanism of PTX nanomicelle by inducing ICD, which can promote antigen presentation by dendritic cells (DCs) and activate antitumor immunity. Notably, nanomicelle encapsulation protected the ICD effects and immune activation, which were hampered by immune system impairment caused by chemotherapy. Compared with traditional formulations, a low dose of nanomicelle-encapsulated PTX (nano-PTX) treatment induced immune-dependent tumor control, which increased the infiltration and function of both T cells and DCs within tumors. However, this antitumor immunity was hampered by highly expressed PD-1 on tumor-infiltrating CD8^+^ T cells and upregulated PD-L1 on both immune cells and tumor cells after nano-PTX treatment. Combination therapy with a low dose of nano-PTX and PD-1 antibodies elicited CD8^+^ T cell-dependent antitumor immunity and remarkably improved the therapeutic efficacy.

**Conclusions:** Our results provide systemic insights into the immune-regulation ability of PTX to induce ICD, which acts as an inducer of endogenous vaccines through ICD effects, and also provides an experimental basis for clinical combination therapy with nano-PTX and PD-1 antibodies.

## Introduction

Chemoimmunotherapy is a prospective strategy to treat cancer 1, 2. The general foundation for this combination is that chemotherapeutic drugs can directly kill tumors via their cytotoxic effect and release tumor-associated antigens that activate the immune system. However, the complicated tumor immune microenvironment and the change caused by chemotherapy may hinder the therapeutic efficacies 3. Furthermore, indiscriminate cytotoxicity may also be a limitation as chemotherapy kills not only tumor cells but also immune cells 4. Paclitaxel (PTX), one of the most effective traditional chemotherapeutical agents used for the clinical treatment of cancer, has multiple immune modulation abilities by selectively decreasing regulatory T cell (Treg) populations 5, promoting calreticulin (CRT) transduction to enhance vaccine effect 6, and inhibiting myeloid-derived suppressor cells (MDSCs) and chronic inflammation in the spontaneous melanoma model 7. Low-dose PTX also directly increases antigen presentation by dendritic cells (DCs) in an interleukin (IL)-12-dependent manner 8. However, the immune regulation mechanisms of PTX are not fully understood.

Based on the severe side effects of chemotherapeutic agents in clinical applications, recent progress in the field of nanotechnology provides a safe and precise delivery system for these cytotoxic drugs. Compared with traditional formulations, PTX coated with nanomicelle has not only better antitumor efficacy but also less systemic toxicity 9-11. Moreover, recent studies have suggested that nanotechnology can dramatically enhance the safety and therapeutic effects of immunotherapy 12-15. Whether and how nanomicelle affect the immune regulation of chemotherapy is of interest in chemoimmunotherapy.

Effective DC activation is key to the initiation of the antitumor immune cycle 16. Immunogenic cell death (ICD) is a specific cell death procedure that involves a series of changes in cell surface proteins and the release of soluble mediators, which operate on phagocytes to initiate the presentation of tumor antigens to tumor killer cells such as DCs, macrophages, natural killer (NK) cells, and T cells 17-19. ICD makes the tumor cells “visible” to the immune system; in particular, phagocytosis by DCs induces a strong antitumor response 17, 19. Tumor cells undergoing ICD upregulate the expression of CRT on the cell surface, which sends “eat me” signals, as well as enabling the phagocytosis of the dying cells by DCs 19, 20. The secretion of high-mobility-group box 1 (HMGB1) and ATP also contributes to ICD progression by promoting DC chemotaxis, antigen presentation, and T cell activation 21-23. Several chemotherapeutic drugs induce ICD, including oxaliplatin (OXP) and anthracyclines, while others, such as cisplatin (CDDP), do not have this effect 19, 24.

Here, we demonstrated a pivotal immune regulation ability of PTX through inducing ICD in several cancer cells. Furthermore, nano-PTX can improve the ICD effects *in vivo* and exert good tumor-control effect. We also provide evidence that PTX treatment increases programmed cell death-ligand 1 (PD-L1) expression within the tumor microenvironment; combination therapy with nano-PTX and PD-1 antibody effectively suppresses tumor growth and prolongs overall survival of tumor-bearing mice. The results of this study suggest a new immune regulation mechanism of PTX, which may be augmented by the nanomicelle package to facilitate immunotherapy.

## Materials and Methods

### Mice and cell lines

Six-week-old female BALB/c-nude, BALB/c, and C57BL/6 mice were purchased from Beijing HFK Bioscience Co. Ltd., Beijing, China. Mouse cell lines including colon carcinoma (CT26), mammary carcinoma (4T1), lung carcinoma (LL/2, LLC1), and melanoma (B16-F10), as well as human cell lines including colon carcinoma (HCT116), mammary carcinoma (MDA-MB-231), and cervical cancer (HeLa) were purchased from American Type Culture Collection (ATCC). CT26-RFP was constructed by lentiviral infection expressing red fluorescent protein (RFP). Mouse MC38 colon cancer cells were provided by Innovent Biologics, Inc. (Suzhou, Jiangsu, P.R. China). Mouse ID8 ovarian cancer cells were provided by Professor Xia Zhao (West China Second University Hospital, Sichuan University, Chengdu, China).

### Drugs and antibodies

For chemotherapeutic drugs, CDDP was purchased from Hanson Pharma, Inc. (Lianyungang, Jiangsu, P.R. China); OXP was purchased from Hengrui Medicine, Inc. (Lianyungang, Jiangsu, P.R. China); and PTX was purchased from TAIJI Industry (Group), Inc. (Chengdu, Sichuan, P.R. China). PTX entrapped with methoxy-poly (ethylene glycol)-*b*-poly (D, L-lactide) (mPEG-PDLLA) was generated as in our previous study 11 and was produced by Guangdong Zhongsheng Pharmaceutical Co., Ltd. China. Briefly, 30 mg of PTX and 150 mg of MPEG-PDLLA were weighed separately and co-dissolved in 2 mL of acetonitrile. Then the solution was evaporated at 37 °C on a rotary apparatus until dry. Therapeutic mouse PD-1 antibodies were provided by Innovent Biologics, Inc.

### Phagocytosis assay

Bone marrow-derived dendritic cells (BMDCs) were isolated from the bone marrow after 8 ~ 10 days of differentiation with granulocyte-macrophage colony-stimulating factor (20 ng/mL, Sino Biological) and IL-4 (10 ng/mL, Sino Biological). Macrophages were purified from tumor tissues with Anti-F4/80 MicroBeads (130-110-443, Miltenyi Biotech Inc.). BMDCs and macrophages were stained with the CFSE Cell Division Tracker Kit (423801, BioLegend). CT26-RFP cells were treated with drugs as indicated for 4 h and then added to the CFSE-labeled BMDC or macrophage culture at a 1:1 ratio. For flow cytometry, the cells were harvested and tested on a NovoCyte flow cytometer. BMDCs and macrophages that phagocytosed CT26 cells were CFSE and RFP-double-positive. For immunofluorescence, cells were fixed in 4% paraformaldehyde. Cell nuclei were stained with DAPI. The concentrations of IL-1β (EMC001b, Neobioscience), IL-12 (EMC006, Neobioscience), IL-18 (EMC011, Neobioscience), and CXCL9 (EK0733, BOSTER) in the supernatant after 24 h of co-culture were quantified using the ELISA Kit.

### Vaccine assay

For the protective assay, CT26 cells were treated with CDDP (150 μM), OXP (300 μM), and PTX (75 μM) for 24 h and harvested. Next, 2 × 10^6^ drug-treated or freeze-thawed (control) cells were inoculated subcutaneously in the left flank of BALB/c mice and 5 × 10^5^ CT26 cells on the right flank 1 week later.

For the therapeutic assay, 5 × 10^5^ CT26 cells were injected subcutaneously on the right flank of BALB/c mice to establish a tumor model. Next, 2 × 10^6^ freeze-thawed (control) or drug-treated cells as indicated were inoculated subcutaneously on the left flank of mice on days 3, 6, 9, and 16 after inoculation. Tumor size was measured with a digital caliper every second day and terminated when the tumor volume exceeded 2,000 mm^3^.

### Tumor model and treatment

To establish subcutaneous tumor models, 5 × 10^5^ CT26 cells and MC38 cells or 2 × 10^5^ 4T1 cells in 100 μL of serum-free medium were injected subcutaneously on the right flank of mice. The tumor size was measured with a digital caliper every other day and terminated when the tumor volume exceeded 2,000 mm^3^. To establish an intraperitoneal transplantation tumor model, 5 × 10^6^ ID8 cells in 500 μL of serum-free medium were injected into the abdominal cavity of mice. The tumor size was determined by the formation of ascites and body weight.

For mono-treatment in subcutaneous CT26 tumor models, PTX or nano-PTX (10 mg/kg) was administered every 2 days for a total of five doses when the tumor volume was 50 ~ 100 mm^3^. For combined treatment in subcutaneous CT26, MC38, and 4T1 tumor models, nano-PTX (10 mg/kg) was administered every 2 days for a total of three doses when the tumor volume was 50 ~ 100 mm^3^, with PD-1 antibody (i.v. 100 μg per mouse) injected every 2 days for a total of three doses after the nano-PTX treatment. In the ID8 tumor model, the same course of treatment was followed starting 1 month after tumor cell inoculation. All experiments were performed in accordance with the Animal Care and Use Committee of West China Hospital, Sichuan University, China.

### Flow cytometry

The tumors were harvested on the sixth day after the last administration, minced, and digested in RPMI-1640 medium containing collagenase IV (0.1%, Gibco), nuclease, and 1% fetal bovine serum at 37 °C for 40 ~ 60 min, after which the cell suspensions were filtered. Fixable Viability Stain 620 (FVS620, 564996, BD Biosciences) was used to discriminate live or dead cells; the cells were then blocked with Fc-block (553142, BD Biosciences) and stained with antibodies. Nuclear factors were permeabilized using a FoxP3 Fixation and Permeabilization Kit (00-5521-00, Invitrogen), while intracellular cytokines were permeabilized using a Fixation/Permeabilization Kit (554714, BD Biosciences) and detected with antibodies. Data were acquired on a NovoCyte flow cytometer. A representative flow gating scheme was shown in Figure S11.

Detailed antibodies, apoptosis assay, western blot, R-T PCR, immunofluorescence, analysis of HMGB1 and ATP release, immunohistochemical staining, and T cell depletion assay are described in the Supplementary materials and methods.

### Statistical analysis

The data were analyzed using GraphPad Prism version 6. Statistical significance was analyzed using unpaired t-tests (two groups) or an one-way analysis of variance (three or more groups). A two-way ANOVA analysis, Huynh-Feldt correction and Tukey's range test were used to analyze tumor volumes. Animal survival is presented using Kaplan-Meier survival curves and analyzed via Log-rank (Mantel-Cox) test. P < 0.05 was considered statistically significant. The figures use the following symbols: * P < 0.05, ** P < 0.01, *** P < 0.001, and **** P < 0.0001, ns (no statistical significance).

## Results

### Low-dose nano-PTX exerts immune-dependent tumor control

**Figure 1:**
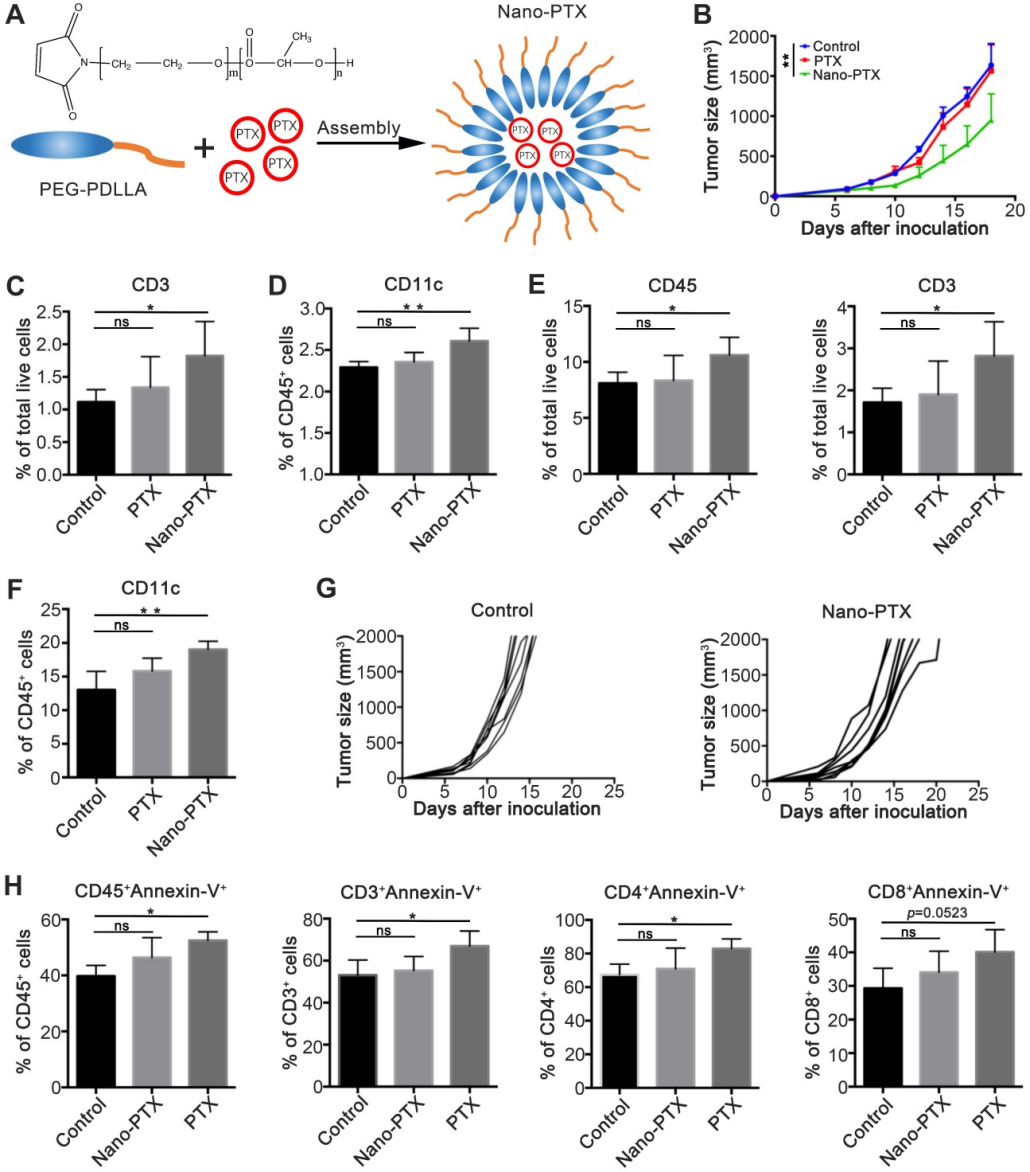
** PTX embedded with nanomicelle exerts immune depended tumor control. A** Schematic representation of PEG-PDLLA-paclitaxel nanomicelle. **B-F** Mice bearing CT26 were treated using PTX (10 mg/kg) embedded with or without nanomicelle for five times and the tissue was harvest on day 20. **B** The tumor growth after treatment, n = 5 mice per group. **C** The percentage of T cells (CD3^+^) in PBMC after treatment, n = 4 mice per group. **D** The percentage of CD11c^+^ cells in draining lymph node after treatment, n = 4 mice per group. **E** The percentage of total immune cells (CD45^+^) and T cells (CD3^+^) in tumor after treatment, n = 4 mice per group.** F** The percentage of dentritic cells (CD11c^+^) in tumor after treatment, n = 4 mice per group. **G** CT26 tumor growth in immune deficiency mice after nano-PTX (10 mg/kg) treatment with low dose, n = 8 mice per group. **H** The percentage of apoptotic immune cells in peripheral blood on day 1 after treatment, n = 4 mice per group. Mean ±SEM was shown. * P < 0.05, ** P < 0.01, ns (no statistical significance).

In our previous studies 11, we constructed a targeted nano-system based on mPEG-PDLLA for reducing the cytotoxicity of PTX (Figure 1A). In this study, we explored the immune regulation effects of nano-PTX. To this end, we established a mouse CT26 colon cancer model, and treated tumor-bearing mice with a low dose of nano-PTX (10 mg/kg). The results showed that, compared with the lack of therapeutic effect of PTX without nanomicelle, nano-PTX significantly delayed tumor growth (Figure 1B). Then, we detected the percentage of immune cells after treatment, which demonstrated that the increase in both T cells (CD3^+^) in peripheral blood mononuclear cells (PBMCs) and DCs (CD11c^+^) in draining lymph nodes was only present in mice treated with nano-PTX (Figure 1C-D). Further analysis of tumor-infiltrating immune cells revealed that the proportions of DCs, total immune cells (CD45^+^), and T cells (CD3^+^) increased after nano-PTX treatment (Figure 1E-F). These results indicate that the antitumor ability of nano-PTX used at low doses might be mediated by the immune system, as the tumor-control ability was abolished when using immune-deficient mice to establish the CT26 tumor model and treating them with the same dose of nano-PTX (10 mg/kg) (Figure 1G).

We speculate that these variant antitumor effects between PTX and nano-PTX can be attributed to the low cytotoxicity of nano-PTX, which is also a benefit of nanomaterial-based therapy 25, 26. To test this, we analyzed apoptotic immune cells in PBMCs after treatment. Results demonstrated that PTX remarkably increased apoptosis of total immune cells (CD45^+^) and T cells (Figure 1H), indicating that PTX treatment attenuated the immune system and subsequent antitumor immunity. Finally, in order to explore an optimal dose of nano-PTX to achieve both immune activation and lower immune system toxicity, we detected the apoptotic level of immune cells after two doses of nano-PTX treatment (10 mg/kg and 40 mg/kg). As shown in Figure S1, except for the lack of apparent differences in tumor- draining lymph nodes (Figure S1A), the percentages of apoptotic overall immune cells, CD4^+^ T, and CD8^+^ T cells remarkably increased in both PBMCs and tumor-infiltrating immune cells after high-dose nano-PTX (40 mg/kg) treatment (Figure S1B-C), while the dose of 10 mg/kg was safe for the immune system (Figure S1B-C).

These results indicate that the nanomicelle package allows PTX to exert immune-regulation ability and induce immune-dependent tumor suppression.

### Nano-PTX treatment increases immune cell infiltration and activation

**Figure 2:**
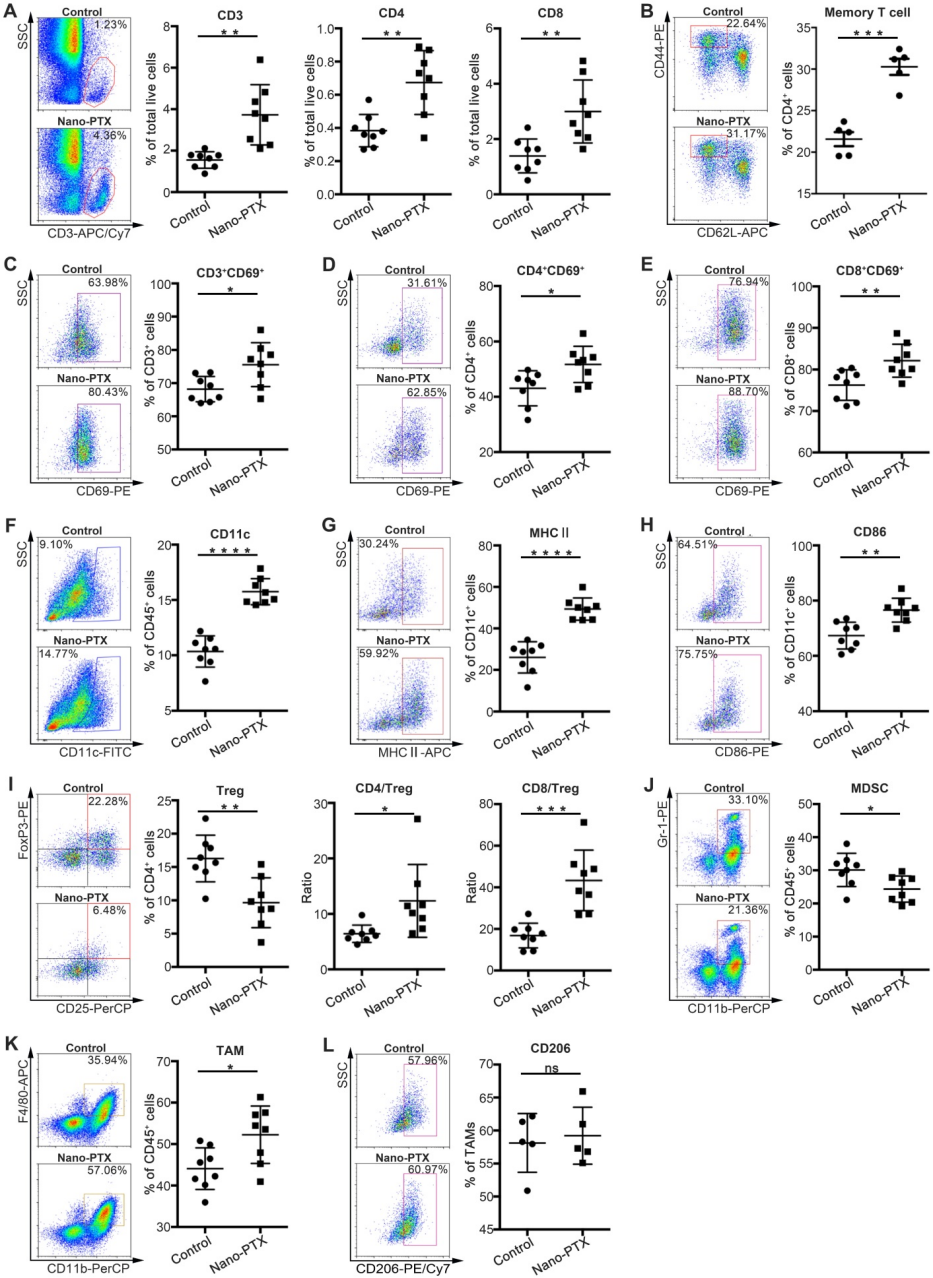
** Nano-PTX treatment increases immune cells infiltration and activation within tumor.** Mice with established CT26 tumors were treated with nano-PTX (10 mg/kg) as described in Figure 5G. Tumor cells were harvested and analyzed by flow cytometry on day 20 (n = 8 mice per group).** A-B** The percentage of T cells (**A**) and memory T (CD4^+^CD44^high^CD62^low/-^) cells (**B**) were shown. **C-E** The activation status of T cell measured by CD69 expression. **F-H** The percentage of DCs (CD11c^+^) (**F**) and its activation status determined by the expression of MHCII (**G**) and CD86 (**H**). **I** The percentage of Tregs (CD4^+^CD25^+^FoxP3^+^) within tumors, and the ratios of CD4^+^ to Tregs and CD8^+^ to Tregs were shown.** J** The percentages of MDSCs. **K-L** The proportion of TAMs (CD11b^+^F4/80^+^) and M2 (CD11b^+^F4/80^+^CD206^+^) TAMs within tumors. Representative flow data was shown in left. Mean ±SEM was shown. * P < 0.05, ** P < 0.01, *** P < 0.001,**** P < 0.0001, ns (no statistical significance).

To investigate the detailed antitumor immunity mechanisms of nano-PTX, we analyzed the immune cell profiles within the tumors and draining lymph nodes after low-dose nano-PTX treatment. Given the important roles of T cells in antitumor immunity 27, we measured T cell infiltration of the tumors via immunohistochemistry. PTX treatment promoted total T cell, CD4^+^ T cell, and CD8^+^ T cell infiltration into the tumors (Figure S2A-C). Flow cytometry analysis further confirmed the increased percentages of total T cell, CD4^+^ T cell, and CD8^+^ T cell infiltration (Figure 2A). Increased percentages of T cells were also observed in draining lymph nodes (Figure S2D). We also found that nano-PTX treatment increased the percentage of memory T cells in spleen, suggesting the formation of immune memory (Figure 2B). Furthermore, these infiltrating T cells in tumor had enhanced activation levels, as indicated by their increased CD69 expression (Figure 2C-E).

DC infiltration is another key factor in antitumor immune response 16. In our study, nano-PTX treatment increased the percentage of DCs in the tumors (Figure 2F). Increased levels of the maturation markers major histocompatibility complex (MHC) II and CD86 (Figure 2G-H) were also observed in draining lymph nodes (Figure S2E-G).

Tregs are a key limiting factor for immune response 28. A decreased percentage of Tregs was observed after treatment, leading to increased ratios of CD4^+^ T cells to Tregs and CD8^+^ T cells to Tregs (Figure 2I). Furthermore, PTX treatment decreased the percentages of immune-suppressive MDSCs (Figure 2J). We also observed an increased percentage of tumor-associated macrophages (TAMs) (Figure 2K), which might be attributed to the upregulation of cytokines related to TAM recruitment, resulting in enhanced tumor cell phagocytosis and antigen presentation 29. Moreover, nano-PTX didn't change the percentage of M2 phenotype TAM defined as CD206 expression (Figure 2L).

These results indicate that nano-PTX treatment changed the immune balance to facilitate antitumor immunity by increasing T cell and DC infiltration and activation, and decreasing immune-suppressive cells.

### PTX induces ICD

**Figure 3:**
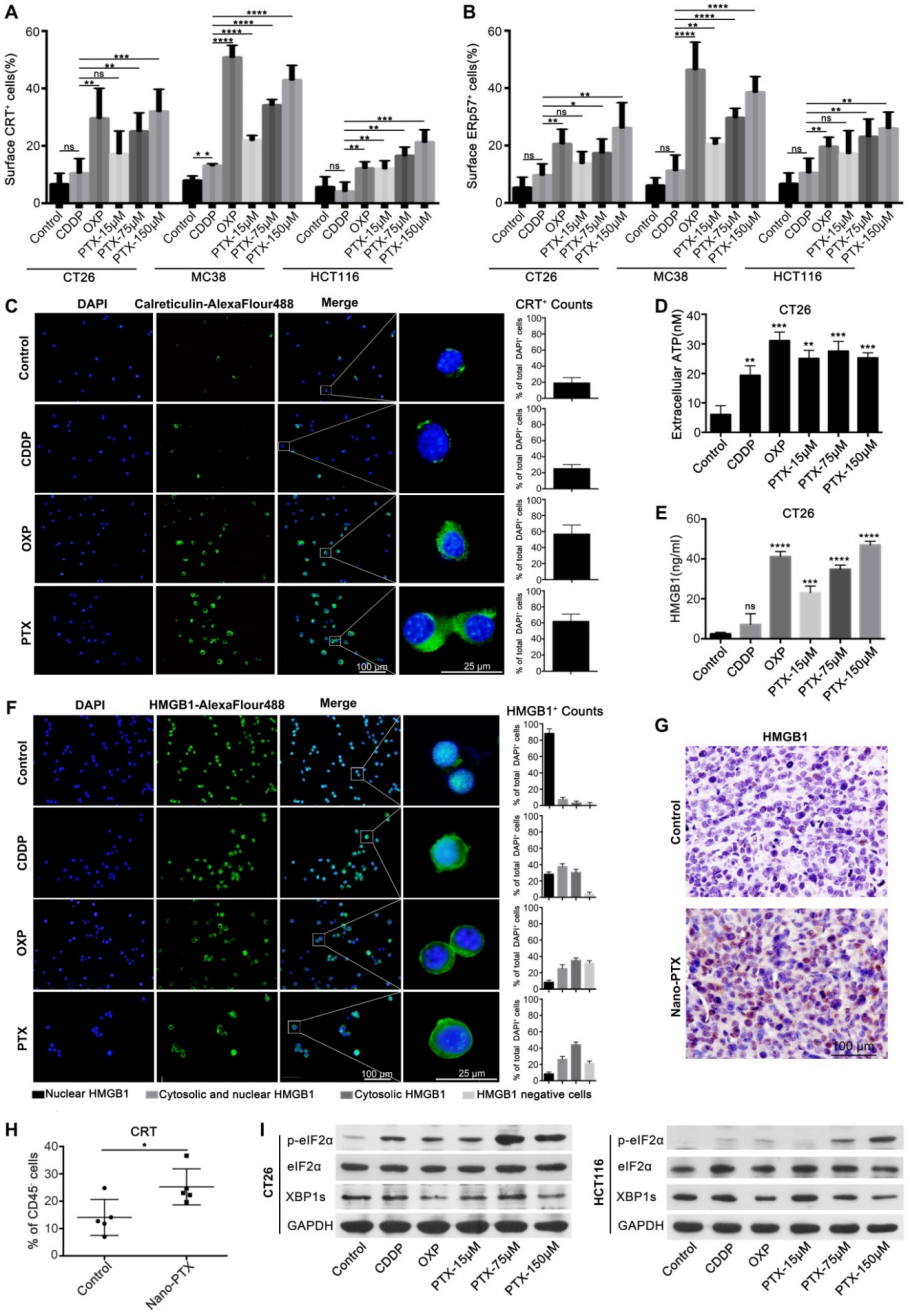
** PTX induces immunogenic cell death. A-B** Flow-cytometry analysis of CRT (**A**) and ERp57 (**B**) on CT26, MC38 and HCT116 cells treated with CDDP (150 µM), OXP (300 µM), and PTX, n= 3 replicates. **C** CRT exposure on the surface of CT26 cells was assessed after short-term stimulation (4 h) with CDDP (150 µM), OXP (300 µM), and PTX (75 µM) treatments by immunofluorescence staining (left panel), statistics was shown in right panel. **D-E** CT26 cells were treated for 24 h *in vitro* and supernatant was collected for detecting the release of ATP (**D**) and HMGB1 (**E**) , n = 3 replicates. **F** Immunofluorescence staining of HMGB1 secretion in CT26 cell after treatment (24 h), statistics was shown in right panel. **G** Immunohistochemistry staining of HMGB1 within CT26 tumor after PTX injection (scale bar, 100 µm). **H** Flow-cytometry detection of CRT on CD45^-^ cells within CT26 tumor after nano-PTX injection, n = 5 mice per group. **I** Western blot showed the expression of protein related to ER stress signaling pathway in CT26 and HCT116 cells after treatment for 4 h. Mean ±SEM was shown. * P < 0.05, ** P < 0.01, *** P < 0.001, **** P < 0.0001, ns (no statistical significance).

The immune-regulation effects of PTX are complicated or paradoxical, with both positive and negative effects on antitumor responses 5, 30, 31. In Figure 2, we show that nano-PTX can increase the number of T cells and DCs. Given the fact that most chemotherapeutic drugs can promote immune responses through inducing ICD effects and DC activation is a key step in starting the antitumor immune cycle 20, we questioned whether PTX could induce ICD effects. To test this, we first analyzed the effects of PTX on colorectal, breast, lung, and melanoma cancer cells. OXP, which induces ICD 24, was used as a positive control in this study. CDDP was used as a negative control 24, 32. We observed that the CT26 cells underwent cell apoptosis in response to CDDP, OXP, and PTX, which was detected by flow cytometry (Figure S3A). The distinctive features of ICD are CRT expression on the cell surface and translocation of the endoplasmic reticulum (ER)-associated protein ERp57 from the ER lumen to the plasma membrane 19, 20, 33. Our results suggest that OXP and PTX but not CDDP induce the translocation of CRT and ERp57 to the cell membrane in mouse cancer cell lines, including colorectal cancer CT26 and MC38, breast cancer 4T1, and lung cancer LL/2; moreover, these effects are dose-dependent for PTX (Figure 3A-B, Figure S3B-D). In addition, the ICD effect induced by PTX was also observed in the HCT116 human colorectal cancer cell line (Figure 3A-B). Immunofluorescence analysis further confirmed that PTX triggered CRT translocation to the cell surface (Figure 3C).

Immunogenic release of ATP and HMGB1 from dying cells is another essential marker of ICD that can promote antitumor immune response 21, 23. We detected increased ATP in the supernatant of CT26 (Figure 3D) and MC38 cells (Figure S3E) after PTX and OXP treatment. Similar results were observed for HMGB1 in CT26 (Figure 3E-F) and MC38 cells (Figure S3F), and also observed a dose-dependent effect for PTX treatment. As ATP and HMGB1 release is a consequence of cell death, increased ATP and HMGB1 were observed after CDDP treatment in this study, consistent with the findings of other studies 24, 34. Moreover, HMGB1 was previously identified as an important marker for ICD *in vivo* 21. Finally, we questioned whether the ICD induced by PTX existed *in vivo*. Mice bearing CT26 tumors were treated with CDDP, OXP, and nano-PTX, and the tumors were harvested for immunohistochemical analysis of HMGB1 expression. PTX treatment increased HMGB1 expression in tumor tissues, and similar results were also found in the OXP-treated group (Figure 3G and Figure S3H). More importantly, upregulated CRT expression in tumor tissues was only observed in CD45^-^ cells (Figure 3H), most of which were tumor cells, but not in CD45^+^ immune cells (Figure S3G), suggesting that PTX is an ICD inducer *in vivo*.

Collectively, these data demonstrate that PTX can induce ICD, which is characterized by the pre-apoptotic exposure of CRT and ERp57 at the cell surface and the release of ATP and HMGB1.

### PTX triggers ER stress response

As CRT and ERp57 translocation is the consequence of the ER stress response induced by drug treatment 2, 21, 35, we next investigated the fate of tumor cells undergoing ICD. Unfolded protein response (UPR) played an important role in determining the fate of tumor cells when undergoing ER stress, which induced apoptosis by upregulation of p-eIF2-α expression and promoted cell survival by activating inositol-requiring kinase to increase XBP1 expression 35-37. Our results suggest that, together with the non-ICD inducer CDDP, both OXP and PTX treatment augmented p-eIF2-α expression in mouse and human tumor cells (Figure 3I and Figure S4A), indicating that the UPR pathway was activated after treatment. To further explore the apoptotic fate of tumor cells responding to ER stress, we analyzed the expression of genes related to this apoptotic process. Our results showed significantly increased mRNA expression of the pro-apoptotic genes *ATF4*, *BBC3*, *BAX*, *BAK1*, and *DDIT3* after treatment in CT26 cells (Figure S4B), while the XBP1 protein and HSPA5 mRNA were attenuated (Figure 3I and Figure S4A-B), which was consistent with previous report 38, 39. Similar findings were also observed in MC38 tumor cells (Figure S4A and Figure S4C). Thus, these results indicate that PTX could trigger the ER stress response, resulting in cell apoptosis.

### PTX treatment facilitates tumor phagocytosis by DCs and macrophages

**Figure 4:**
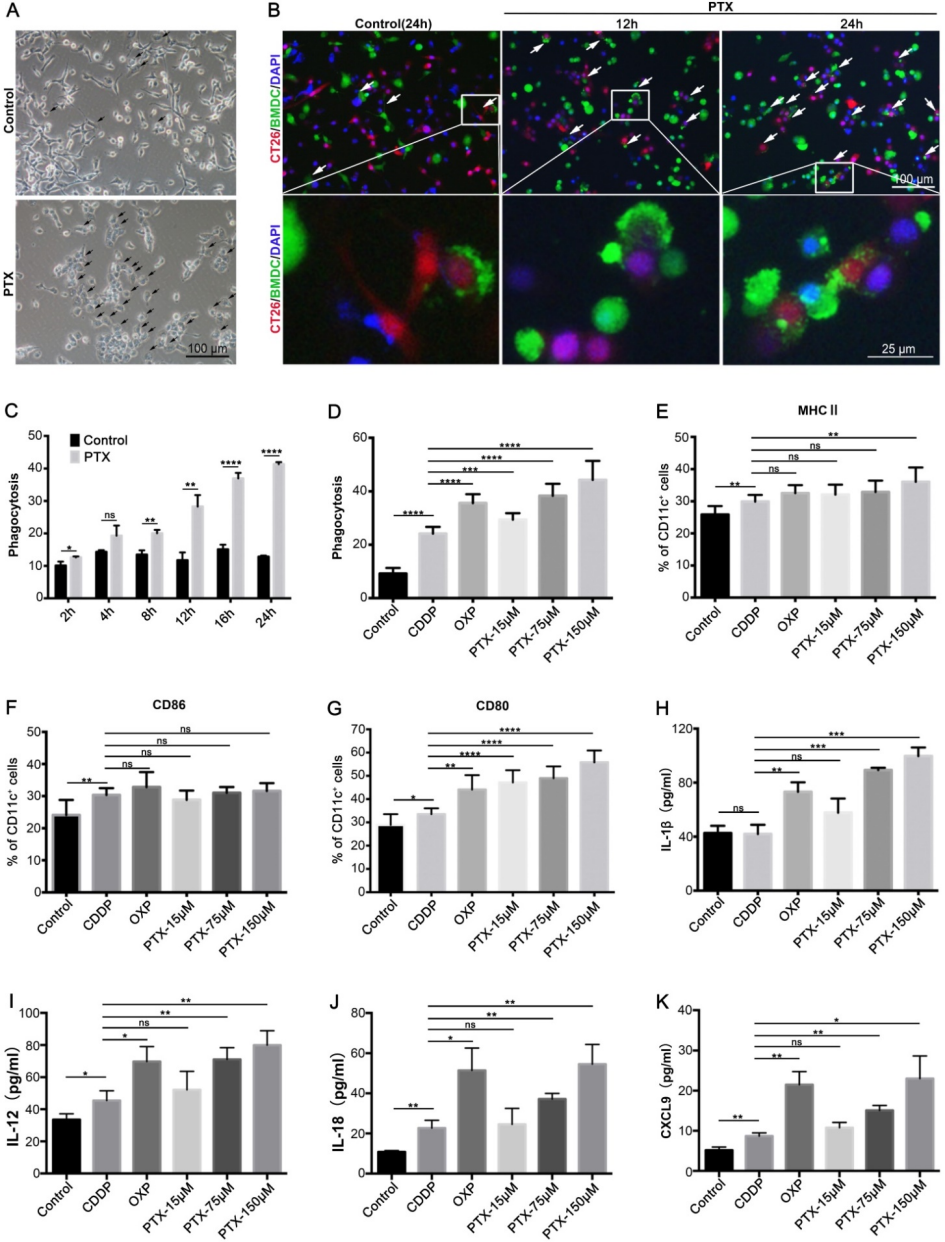
** PTX-treated tumor cells are readily phagocytosed by BMDCs. A** Representative image of co-culture untreated (Control) or PTX-treated CT26 cells with BMDCs, the black arrow indicated the dying cell was phagocytosed by BMDCs (Scale bars, 100 µm). **B** Representative images of co-culture BMDCs (green) with PTX-treated CT26-RFP cells (red) at different time points, the white arrow indicated phagocytosis (Scale bar, 100 µm). An enlargement area was showed below (Scale bar, 25 µm). **C-D** The quantification of CT26 (RFP) cells phagocytosed by BMDCs (CFSE), n = 3 replicates. **E-K** CT26 cells were treated with CDDP (150 µM), OXP (300 µM) and PTX for 4 h and then co-cultured with BMDCs for an additional 24 h. The expression of MHCII (**E**), CD86 (**F**), and CD80 (**G**) on BMDCs was assessed by flow cytometry, n = 7 replicates; IL-1β (**H**), IL-12 (**I**), IL-18 (**J**), and CXCL9 (**K**) in the co-culture supernatant were determined by ELISA assay, n = 3 replicates. Mean ±SEM was shown. * P < 0.05, ** P < 0.01, *** P < 0.001, **** P < 0.0001, ns (no statistical significance).

ICD enhances the immunogenicity of tumor cells, making tumor cells visible to the immune system, especially to DCs 19, 20. Thus, we evaluated whether PTX treatment could make tumor cells more susceptible to phagocytosis by DCs. BMDCs were isolated from the bone marrow (Figure S5A-C). CT26 cells expressing RFP were treated with PTX for 4 h and then co-cultured with CFSE-labeled BMDCs. The results suggest that PTX treatment increases BMDC phagocytosis, which was characterized by increased BMDC chemotaxis around the tumor cells and increased tumor cell signals within BMDCs (Figure 4A-B). Further analysis by flow cytometry showed an increased percentage of BMDCs phagocytosing tumor cells over time (Figure 4C), comparable to the effects observed in the positive control (OXP) (Figure 4D). We also observed increased DC maturation after treatment based on MHC II, CD86, and CD80 expression (Figure 4E-G). However, increased phagocytosis and DC maturation were observed in CDDP treatment; considering the intrinsic ability of DCs to eliminate dead cells, we also investigated the antigen presentation abilities of DCs after treatment, which are key factors for activation of the immune response. IL-1β, IL-12, IL-18, and CXCL9 secreted by DCs are essential for antigen presentation 8, 40, 41 and T cell chemotaxis 42; our data show that PTX and OXP but not CDDP treatment significantly increased IL-1β, IL-12, IL-18, and CXCL9 expression in the co-culture supernatant (Figure 4H-K), indicating that PTX treatment preferred the tumor cells presented by DCs *in vitro*. Similar increases in phagocytosis and IL-1β secretion were also observed in macrophages purified from tumor tissues and co-cultured with PTX-treated tumor cells (Figure S6A-D). Taken together, these results demonstrate that PTX-treated tumor cells were easily phagocytosed and presented by DCs and macrophages, suggesting their potential to activate an antitumor immune response *in vivo*.

### PTX-prepared cell vaccines show both prophylactic and therapeutic effects

**Figure 5:**
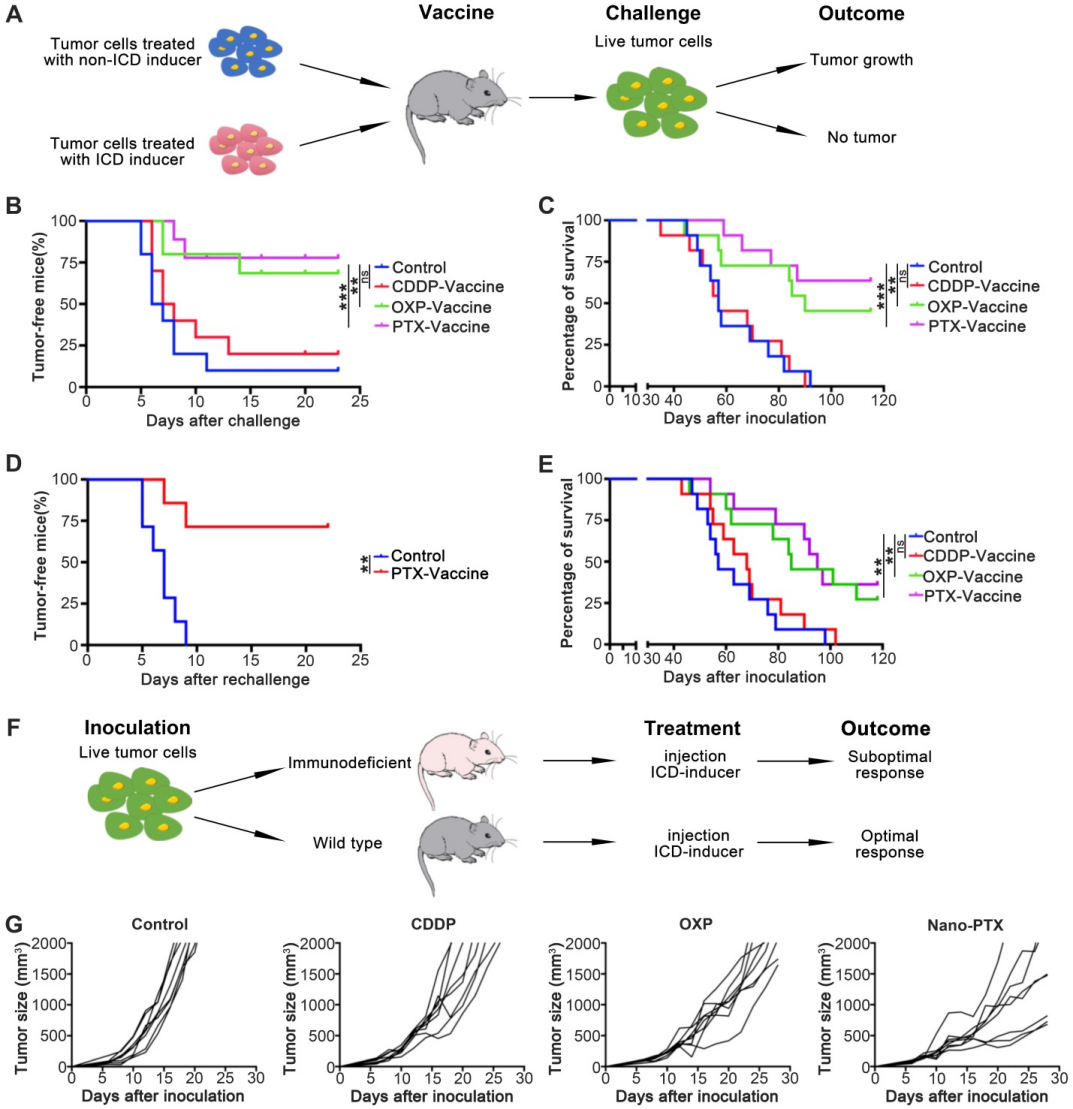
** PTX treatment generates immune dependent tumor suppression. A** Treatment schedule for vaccine assay. **B-E** CT26 cell vaccines were prepared by treating tumor cells with CDDP (150 µM), OXP (300 µM), and PTX (75 µM) for 24 h. **B** The tumor formation of mice receiving protective vaccination, n = 10 ~ 11 mice per group. **C** The overall survival of mice receiving protective vaccination, n = 10 ~ 11 mice per group. **D** The tumor formation of surviving mice receiving re-challenge with a high dose (1 × 10^6^ cells), n = 7 mice per group. **E** The overall survival in therapeutic vaccination, n = 11 mice per group. **F** Treatment schedule for Figure 1F and (**G**). **G** CT26 tumor growth in immune competent mice after CDDP (1 mg/kg), OXP (5 mg/kg) and nano-PTX (10 mg/kg) treatment with low dose for five times, n = 8 mice per group. ** P < 0.01, *** P < 0.001, ns (no statistical significance).

To investigate the antitumor abilities of ICD induced by PTX, we performed vaccination assays, which are the gold-standard approach to evaluate the effect of ICD *in vivo* 19. First, we injected tumor cell vaccines before CT26 inoculation according to the treatment schedule (Figure 5A); the results suggest that tumor cell vaccines prepared with PTX and OXP rather than CDDP protect mice from tumor formation (Figure 5B) and prolong overall survival (Figure 5C). To test whether this protection was long-term and with immunological memory, we first analyzed the memory T cells after vaccination; the cell vaccines prepared with PTX and OXP increased the percentage of memory T cells (Figure S7A). To further verify this effect, tumor re-challenge assays were performed 1 month later; in these assays, 75% of tumor-free mice completely rejected a re-challenge with a higher dose of tumor cells (1 × 10^6^ cells), proving the long-term and immunological memory effects (Figure 5D). We further investigated the antitumor abilities of ICD in a therapeutic model by injecting the tumor cell vaccines after tumor establishment. We found that, unlike the non-ICD inducer CDDP, the PTX-prepared tumor cell vaccines remarkably prolonged survival (Figure 5E) and delayed tumor growth (Figure S7B-C). Another ICD inducer, OXP, also showed similar effects (Figure 5E and Figure S7B-C). In brief, PTX-prepared tumor cell vaccines were able to produce prophylactic and therapeutic effects by inducing ICD.

We next questioned whether the antitumor effects induced by PTX-treated tumor cell vaccines could be acquired by direct nano-PTX injection (Figure 5F). In immune-competent mice, direct nano-PTX administration (i.v.) showed more effective tumor control and prolonged survival than in the untreated and non-ICD inducer CDDP groups (Figure 5G and Figure S7D). Similar effects were also observed in the group administered OXP (Figure 5G and Figure S7D).

Because the antitumor ability of nano-PTX was abolished in immunodeficient mice (Figure 1G), these data provide strong evidence that direct nano-PTX administration could also produce ICD to transform tumor cells into endogenous vaccines *in situ* and trigger immune system-dependent antitumor effects.

### Nano-PTX treatment enhances PD-L1 expression within the tumor microenvironment

**Figure 6:**
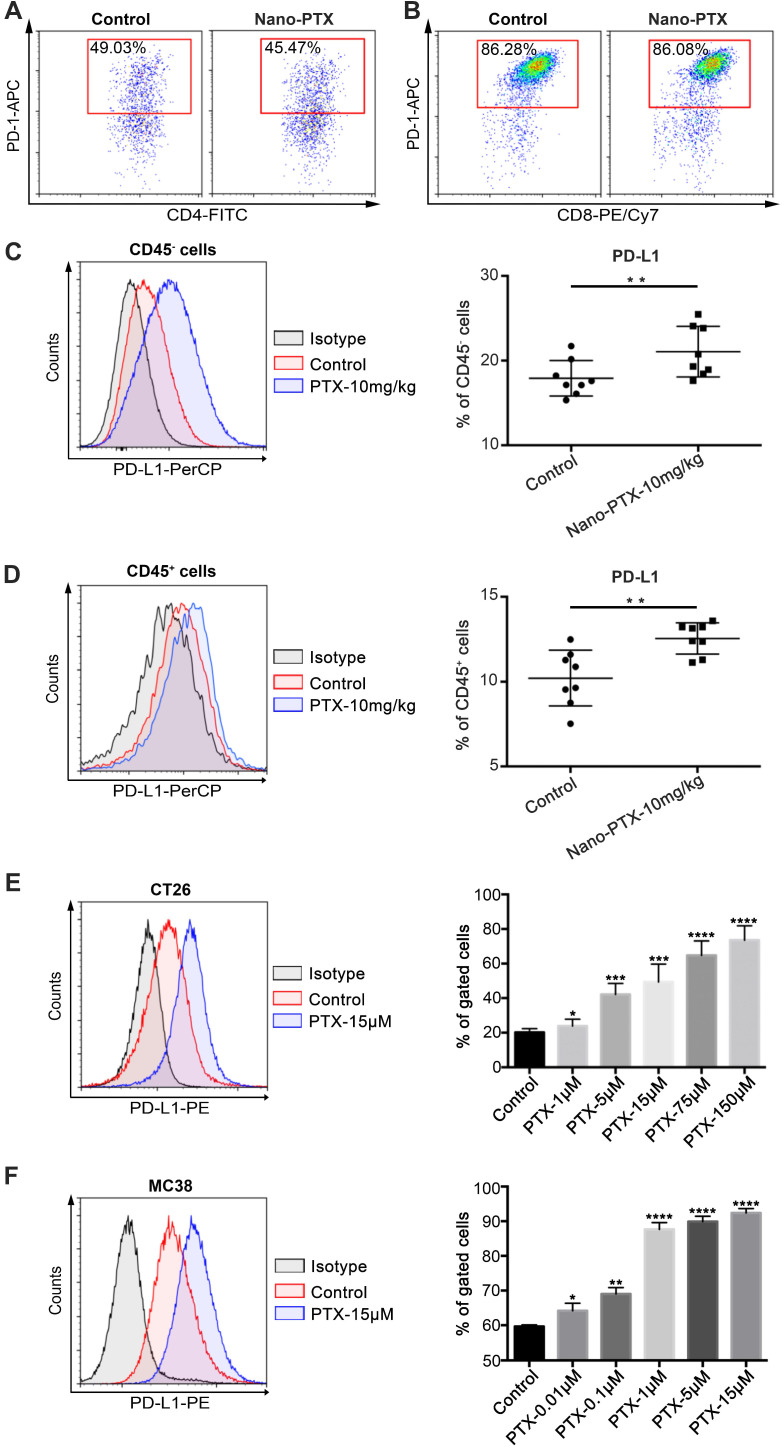
** PTX treatment up-regulates PD-L1 expression within tumor microenvironment. A-D** Mice with established CT26 tumors were treated with nano-PTX as described in Figure 5G, tumors were harvested and analyzed by flow cytometry on day 20. Representative flow data of PD-1 gated on CD4^+^ T cells (**A**) and CD8^+^ T cells (**B**) were shown. The percentages of PD-L1^+^ cells gated on CD45^-^ cells (**C**) and CD45^+^ cells (**D**) were shown, n = 8 mice per group. **E-F** CT26 cells (**E**) and MC38 cells (**F**) were treated with PTX for 24 h in different concentrations as indicated. The PD-L1 expression was detected using flow cytometry, n = 8 replicates. Mean ±SEM was shown. * P < 0.05, ** P < 0.01, *** P < 0.001, **** P < 0.0001.

While we showed that PTX could induce ICD and trigger immune system-dependent antitumor effects, the therapeutic outcomes were less beneficial than we expected. Therefore, we speculate that other immune escape mechanisms in the tumor microenvironment or induced by nano-PTX treatment might limit the antitumor immune response. To verify this hypothesis, we analyzed the expression of immune checkpoint PD-1/PD-L1 in the tumors. PD-1 was highly expressed on tumor-infiltrating T cells, especially on CD8^+^ T cells (Figure 6A-B), and nano-PTX treatment did not increase PD-1 expression (Figure S8A-C). However, nano-PTX treatment enhanced PD-L1 expression on both non-immune (CD45^-^) and immune (CD45^+^) cells in the tumor microenvironment (Figure 6C-D). As tumor cells account for most of the components in non-immune cells (CD45^-^), we explored whether PTX treatment could directly upregulate PD-L1 expression on tumor cells *in vitro*. CT26 tumor cells were treated with different doses of PTX for 24 h; we observed a dose-dependent increase in PD-L1 expression (Figure 6E). Similar results were observed in MC38 tumor cells (Figure 6F). PD-L1 was also constitutively expressed on tumor cells (Figure 6E-F). Collectively, these data indicate that highly expressed PD-1 on T cells and upregulated PD-L1 expression may be the key limitations of ICD-based PTX treatment (Figure 5G).

### Combination of nano-PTX and PD-1 antibody effectively promotes tumor regression and prolongs survival

**Figure 7:**
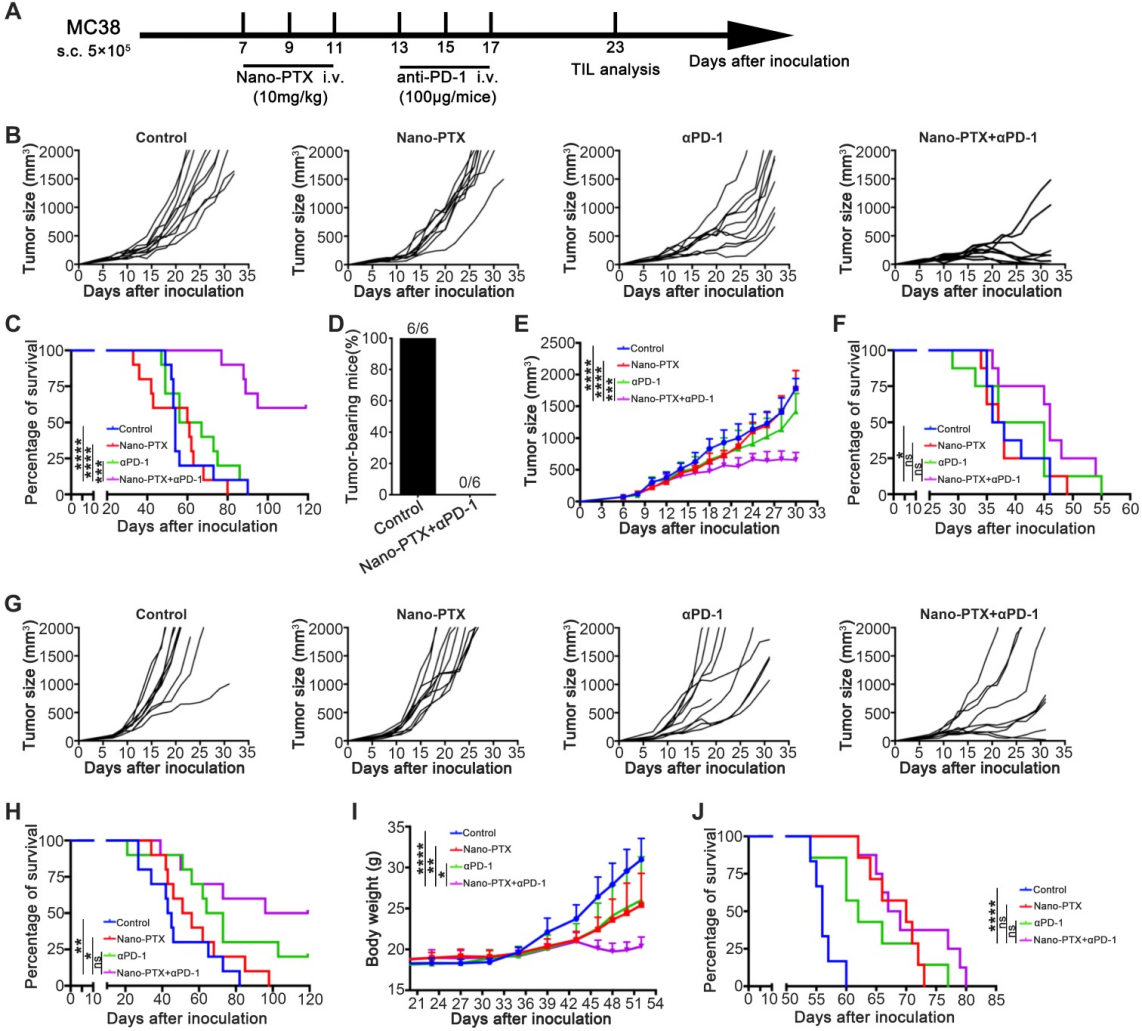
** Combination therapy with paclitaxel and PD-1 antibody induces tumor regression. A-C** Established MC38 subcutaneous tumor model were treated as scheme (**A**). Individual tumor growth of mice (**B**) and the overall survival (**C**) were monitored, n = 9 ~ 10 mice per group. **D** The surviving mice from the combination treatments were re-challenged with a high dose (1 × 10^6^) of MC38 cells 10 weeks later, n = 6 mice per group. **E-F** 4T1 subcutaneous tumor was treated as indicated. The tumor growth curve (**E**) and the overall survival (**F**) were shown, n = 8 ~ 9 mice per group. **G-H** CT26 subcutaneous tumor was treated as indicated. Individual tumor growth of mice (**G**) and the overall survival (**H**) were shown, n = 9 ~ 10 mice per group. **I-J** Established ID8 intraperitoneal transplantation tumor model was treated as indicated. The body weight growth (**I**) and the overall survival (**J**) were shown, n = 6 ~ 8 mice per group. Mean ±SEM was shown. * P < 0.05, ** P < 0.01, *** P < 0.001, **** P < 0.0001, ns (no statistical significance).

Combination therapy has been shown to be an ideal approach to overcome multiple immune-suppressive mechanisms and improve treatment outcomes 43, 44. As the PD-1/PD-L1 pathway was upregulated after PTX treatment, we further tested the efficacy of the combination of nano-PTX and PD-1 antibody (Figure 7A). In the mouse MC38 tumor model, combination therapy remarkably inhibited tumor growth relative to that in the control or monotherapy groups (Figure 7B), leading to complete tumor regression in 78% of the mice (Figure 7B) and prolonged survival of tumor-bearing mice (Figure 7C). To investigate whether this combination therapy could confer lifelong protection, we performed a re-challenge assay 8 weeks after cessation of drug injection. All surviving mice following the combination treatment completely rejected a re-challenge with a higher dose (1 × 10^6^) of MC38 cells (Figure 7D). Regarding the long-term effects, none of the mice had formed a tumor at the end of the experiment and 10 months after re-challenge (data not shown), suggesting effective immunological memory. To extend this combination strategy to other tumors, we established several tumor models to evaluate the effects of this therapy. In a mouse breast cancer (4T1) subcutaneous model, which was resistant to immunotherapy, combination therapy demonstrated higher therapeutic efficacy than monotherapy or the control (Figure 7E and Figure S9A), as well as prolonged survival (Figure 7F). In another mouse colon cancer model (CT26), combination therapy showed antitumor effects, as indicated by delayed tumor growth and prolonged survival (Figure 7G-H). Malignant ascites are closely related to poor prognosis of ovarian cancer 45; we established a mouse ovarian cancer model by intraperitoneal injection of ID8 cells, which produced ascites in the late stages, and evaluated the therapeutic effects of the combination therapy on advanced ovarian cancer. As expected, the combination therapy arrested the increase in body weight caused by ascites production (Figure 7I) and also prolonged the survival (Figure 7J). No abnormal behaviors or body weights were observed during the course of treatment in the MC38 and CT26 models (Figure S9B-C), suggesting low toxicity. Taken together, these data provide evidence that the combination therapy synergistically conferred antitumor effects in multiple tumor models.

### Combination therapy elicits CD8^+^ T cell-dependent antitumor immunity

**Figure 8:**
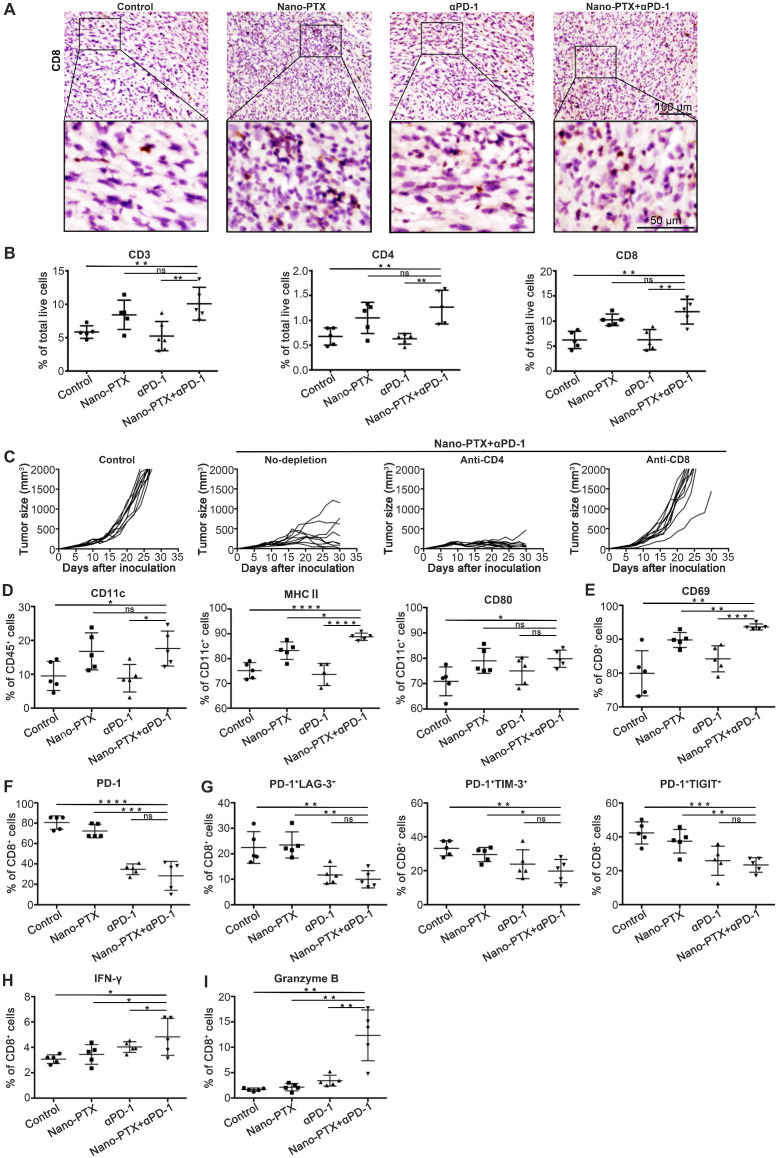
** The combination therapy augments immune cells infiltration and activation.** Mice with established MC38 tumors were treated with nano-PTX and PD-1 antibody as described in Fig 7A, Tumors were isolated on day 23 for analysis. **A** Immunohistochemistry staining of CD8 within MC38 tumor after treatment (scale bar, 100 µm). An enlargement of the squared area was showed below (Scale bar, 50 µm). **B** The numbers of tumor-infiltrating CD3^+^, CD4^+^ and CD8^+^ T cells. **C** Tumor growth with CD4^+^ T cells or CD8^+^ T cells depletion, n = 9 ~ 10 mice per group.** E** CD69 expression on CD8^+^ T cells. **F** PD-1 expression on CD8^+^ T cells. **G** The percentages of PD-1^+^ LAG3^+^, PD-1^+^ TIM-3^+^ and PD-1^+^ TIGIT^+^ double positive CD8^+^ T cells. **H-I** Spleen cells were stimulated with leukocyte activation cocktail (BD Bioscience) containing brefeldin A for 4 h, and the expression of IFN-γ (**H**) and GzB (**I**) was determined by flow cytometry, gated on CD8^+^ T cells. Mean ± SEM was shown. * P < 0.05, ** P < 0.01, *** P < 0.001, **** P < 0.0001, ns (no statistical significance).

To identify the mechanisms responsible for the therapeutic outcome of combination therapy, we initially detected T cell infiltration within tumor tissue by immunohistochemistry. T cell infiltration was higher for combination therapy than in the other groups (Figure 8A, Figure S10A-B). To confirm this result, we further analyzed the T cell profiles in tumors by flow cytometry. The combination treatment boosted the percentage of T cells in the tumor (Figure 8B). The increased T cell infiltration was owing to PTX but not anti-PD-1 treatment (Figure 8B).

To determine which T cell subsets were responsible for tumor control, the CD4^+^ or CD8^+^ T cells were depleted, and the depletion efficacy was confirmed (Figure S10C-D). Mice with CD8^+^ T cell depletion showed complete abrogation of tumor rejection (Figure 8C), indicating that CD8^+^ T cells were required to achieve therapeutic efficacy.

The changes in DCs were similar to those described above for T cells; increased numbers of DCs were observed only in groups treated with PTX, as well as increased expression levels of the maturation markers MHC II and CD86 (Figure 8D). These data emphasize the key role of PTX in inducing ICD effects, followed by DC and T cell activation.

To study the function of T cells in tumors, we detected the expression of activation and co-inhibitory molecules in T cells. Combination therapy significantly increased the expression of the activation marker CD69 (Figure 8E). Previous studies have shown the close relationship between co-inhibitory molecules and T cell dysfunction 46. Decreased PD-1 expression on CD8^+^ T cells was observed after combination treatment (Figure 8F), with significantly decreased numbers of PD-1^+^LAG-3^+^ double-positive CD8^+^ T, PD-1^+^TIM-3^+^ double-positive CD8^+^ T, and PD-1^+^TIGIT^+^ double-positive CD8^+^ T cells (Figure 8G), indicating a severely exhausted status 47. In addition, the numbers of IFN-γ and granzyme B-producing CD8^+^ T cells in the spleen increased after combination treatment (Figure 8H-I).

Thus, these results show that combination therapy with nano-PTX and PD-1 antibody enhanced infiltration of functional DCs and T cells in the tumor microenvironment and triggered an antitumor immune response. It is clear that in this combination regimen, PTX acts as a trigger that induces ICD effects and activates the immune response, while PD-1 antibody releases the immunosuppression mediated by the PD-1/PD-L1 signal within the tumor microenvironment.

## Discussion

Recently, nanocarriers have shown great potential for cancer therapy as powerful delivery systems that improve therapeutic efficacies, with the benefit of low cytotoxic effects on other healthy tissues or organs or prolonged residence time of drugs in the body by slow elimination from the target site 25, 48. For delivery of chemotherapeutic drugs, nanocarriers are emerging as a promising approach to overcome the serious side effects of chemotherapeutic agents that are the main concerns in the clinical application of these agents, other strategies are also well studied, including liposomes 49, micelles 50, albuminbased formulation 51. Recent progresses in the field of nanotechnology-based chemotherapy are focused on optimizing or modifying nanocarriers to enhance their targeting potential and safety, such as deoxycholic acid-modified 52, sodium cholate-modified 53 or peptide-conjugated 54. In our previous studies, we demonstrated that nano-PTX accumulated in tumor tissue and inhibited tumor growth more efficiently than traditional formulations 11, which was consistent with the results of other studies using similar approaches 9, 55. In this study, we found that, even at a low dose (10 mg/kg), nano-PTX could significantly suppress tumor growth, and more importantly, it showed more efficient immune activation, which was responsible for tumor control. In other words, nanomicelle encapsulation augments or protects the immune-activation effects of PTX. These effects were mediated by lower cytotoxicity on the immune system by nano-PTX than by the traditional formulation, because PTX increased the percentage of apoptotic immune cells in the peripheral blood, leading to attenuated antitumor immune responses. Moreover, in a glioblastoma model, local chemotherapy promoted DC and effector T cell infiltration of tumors; in contrast, systemic chemotherapy led to systemic and intratumoral lymphodepletion, and immune memory was also abrogated in the long-term survivors 4. As a consequence, the nanomicelle package conferred on PTX a more effective antitumor ability and better compatibility with the immune system, which could be combined with immunotherapy to further improve outcomes. Of note, emerging evidence has indicated promising combination benefits of nanotechnology and cancer immunotherapy 10, 12, 14, 56-58.

Despite the cytotoxic effects, chemotherapeutic agents can modulate the tumor immune microenvironment and affect immunotherapy efficacy 1. The benefits of combination chemotherapy with immunotherapy have been demonstrated 3, 59. However, the mechanisms by which chemotherapeutic drugs change the tumor and, thus, improve the outcomes of combination therapy are not fully understood. Our present study proposed a modulating mechanism for PTX through inducing ICD effects; these effects could be expanded by nanomicelle encapsulation and sensitization of tumors to anti-PD-1 immunotherapy. Data from our study demonstrated the antitumor abilities of ICD induced by PTX and provided the basis for therapy combining PTX with PD-1 antibody.

ICD can elicit a functional death that can be recognized by the immune system 19. Tumor cells undergoing ICD are more easily phagocytosed by antigen presentation cells and processed for antigen presentation, activating prime T cells and inducing systemic antitumor immune response. One of the most distinctive characteristics of ICD is that it can be “seen” by the immune system through CRT translocation to the cell membrane. Although CDDP can attract myeloid cells into the tumor to foster the stimulation of tumor-specific CD8^+^ T cells 60 and synergize with vaccines to promote tumor cell death 61, it cannot induce the exposure of CRT 24. When combined with CRT protein 24 or ER stress inducers such as thapsigargin or tunicamycin 32, the immunogenicity of cisplatin-induced cancer cell death could be restored. Several antitumor agents that have been successfully used in the clinic for decades, including radiotherapy, doxorubicin, cyclophosphamide, OXP, and cetuximab, are ICD inducers 24, 38, 62. Our study established PTX as a bona fide ICD-inducing agent as validated by measuring the levels of CRT, ERp57, ATP, and HMGB1 in several mouse and human tumor cell lines.

Although the immune-activation ability of ICD is attractive, it may also be a reason for rapid relapse after chemotherapy, because most ICD inducers are cytotoxic agents that produce severe toxicity on the immune system accompanied with tumor killing. Hence, the immune-activation ability of ICD is usually ignored and attenuated in cancer treatment. In this study, we showed that nanomicelle encapsulation could protect the ICD effects by reducing side effects on the immune system, which provided the possibility to simultaneously achieve rapid reduction of tumor burden via direct tumor killing and long-term effects via immune activation in chemotherapy. From another perspective, the ICD effects induced tumor cells as a vaccine *in situ*; a recent study indicated that vaccination *in situ* could achieve objective benefits in clinical settings 63.

Successful immune activation is key for initiation of the antitumor immune cycle 64. However, the tumor microenvironment is armed with multiple immune escape mechanisms to limit antitumor immunity 44, 64, 65. These limitations, which were also found in our study, were preexisting or arose after treatment in the tumor microenvironment. Our results demonstrate that nano-PTX suppresses tumor growth in an immune-dependent fashion, but the efficacies are undesirable. Further analysis revealed that PD-1 was highly expressed on tumor-infiltrating CD8^+^ T cells, and PD-L1 was constitutively expressed on tumor cells. Furthermore, both tumor cells and immune cells in the tumors showed increased PD-L1 expression after PTX treatment, and the number of TAMs also increased after PTX administration. This indicates that, despite PTX inducing ICD and starting the antitumor immune cycle, the antitumor immune responses are hampered at the last step of the immune cycle, in which T cells kill tumor cells.

Accumulating evidence has shown that combination therapy is a promising approach to overcome the intricate limitations in the tumor microenvironment and improve therapeutic outcomes 3, 44. The ability of PTX to trigger ICD and upregulate PD-L1 expression after treatment suggests the potential of combined PTX and PD-1/PD-L1 blockade; from our results, significantly improved effects were observed in several tumor models with the combination of nano-PTX and PD-1 antibody. In this combination strategy, increases in DC and T cell numbers were limited in groups administered nano-PTX, indicating that nano-PTX mainly triggered tumor cells to undergo ICD effects *in situ* and then systematically promoted DC activation and T cell augment and infiltration. Anti-PD-1 treatment reversed T cell suppression and exhaustion mediated by PD-1/PD-L1 signals within the tumor microenvironment. Therefore, this combination strategy can overcome insufficient immune activation in the early stages and immune inhibition in the late stages of the immune cycle that dampen antitumor immunity and can also demonstrate a synergistic antitumor effect.

The choice of dose and timing is an important consideration for the successful combination of chemotherapy and immunotherapy, especially for drugs with cytotoxicity 4, 44. In high doses, PTX can produce indiscriminate cytotoxicity in both tumor and immune cells (DCs, T cells). For example, local but not systemic chemotherapy enhanced the efficacy of anti-PD-1 therapy 4. In this study, a low dose of PTX (10 mg/kg) was administered in sequential doses in the animal experiment. The results showed significant induction of ICD in tumor cells (CD45^-^) but not immune cells (CD45^+^), which indicates the potential for the combination of the tumor-killing effects of PTX and low cytotoxicity in immune cells. However, a higher dose of PTX (40 mg/kg) resulted in significant apoptosis of CD45^+^, CD4^+^, and CD8^+^ cells. Therefore, although the combined effects of high doses of PTX were not tested in this study, we can speculate that they may show a poor synergistic effect with the anti-PD-1 treatment. In addition, it was difficult to investigate immune modulation of PTX-induced ICD that sensitizes tumors to immunotherapy, because we cannot determine whether the therapeutic outcome was mediated by cytotoxicity or immunity when using high doses of PTX. Other studies have also suggested that repeated injection with low-dose chemotherapy could work well with immunotherapy 8.

## Conclusions

Our study demonstrates a key immune-regulation ability of PTX via inducing ICD and generating vaccines *in situ*, which can effectively initiate antitumor immunity. Furthermore, nanocarrier-based PTX delivery could further enhance the ICD effects through targeted delivery and improved compatibility with the immune system, which can be extended to other nanotechnology-based cancer therapies. Our results also expand the mechanistic basis for the combination of nano-therapy, PTX, and anti-PD-1 immunotherapy.

## Supplementary Material

Supplementary materials and methods, figures.
