# Supplementary material for: Nanomicelle protects the immune activation effects of Paclitaxel and sensitizes tumors to anti-PD-1 Immunotherapy
Source: Theranostics. 2020 Jul 9;10(18):8382–99. doi: 10.7150/thno.45391 (PMC7381738; doi:10.7150/thno.45391)
Supplement: Supplementary file 1 — Supplementary materials and methods, figures. [file thnov10p8382s1.pdf]

Supplementary materials and methods

Antibodies

Anti-CD4 (GK1.5) and anti-CD8 (2.43) used for depletion were purchased from BioXCell. Antibodies to CD4 (RM45), CD45 (30-F11), CD11b (M1/70), CD11c (N418), major histocompatibility complex II (MHC II, M5/114.15.2), CD80 (16-10A1), F4/80 (BM8), Gr-1 (RB6-8C5), CD69 (H1.2F3), lymphocyte-activation gene 3 (LAG-3, C9B7W), T cell immunoglobulin-3 (TIM-3, RMT3-23), T cell immunoreceptor with Ig and ITIM domains (TIGIT, 1G9), PD-1 (RMP1-30), PD-L1 (10F.9G2), and granzyme B (GzB, GB11) were purchased from BioLegend. Antibodies to CD3 (17A2), CD8 (53-6.7), CD25 (3C7), CD86 (GL1), forkhead box P3 (Foxp3, R16-715), and interferon gamma (IFN-γ, XMG1.2) were purchased from BD Biosciences.

Apoptosis assay

CT26 cells were cultured in the absence or presence of drugs (cisplatin [CDDP], 150 μM; oxaliplatin [OXP], 300 μM; paclitaxel [PTX], 15, 75, and 150 μM) for 24 h, as indicated. Cell death and apoptosis were assessed by flow cytometry using the Annexin V-FITC/PI Apoptosis Detection Kit (KeyGEN BioTECH, KGA108) and the Annexin V-PE/7AAD Apoptosis Detection Kit (Vazyme, A213-01).

Western blot

Cells were treated with drugs as indicated in serum-free medium for 4 h, followed by collection of the cell lysates. The primary antibodies used to assess ER stress response included anti-phosphorylated eukaryotic initiation factor 2 alpha (anti-p-eIF2-α, 1:1000, 9721, CST), anti-eIF2-α (1:1000, 9722, CST), anti-X-box binding protein 1 (anti-XBP1s, 1:1000, 12782, CST), and anti-glyceraldehyde-3-phosphate dehydrogenase (anti-GAPDH, 1:20000, 60004-1-IG, ProteinTech).

R-T PCR

Total RNA was extracted using TRIzol (Life Technologies). RNA was reverse-

transcribed and mRNA expression was analyzed using a PrimeScript TM RT reagent Kit (TaKaRa) on a LightCycler 96 System (Roche). The gene expression was normalized to housekeeping gene β-actin.

Immunofluorescence detection of CRT and HMGB1

CT26 cells were treated with drugs as indicated for 4 h (CRT detection) or 24 h (high-mobility-group box 1 [HMGB1] detection) and then were fixed with 4% paraformaldehyde. When detecting HMGB1, the cells were permeated with 0.1% Triton X-100 after fixation. The cells were stained with anti-CRT (1:100, ab2907, Abcam) or anti-HMGB1 (1:100, 3935, CST) and then detected by an Alexa Fluor 488-labeled secondary antibody (1:400, 4412, CST). Cell nuclei were stained with 4′,6-diamidino-2-phenylindole (DAPI).

The number of cells with CRT-Alexa Fluor-488 positive signal was counted and quantified from three pictures of every group. For the location of the positive signal, HMGB1-Alexa Fluor-488 was divided into four situations: (a) the signal was detected only in the nucleus; (b) the signal was detected in both the nucleus and cytoplasm; (c) the signal was detected only in the cytoplasm; or (d) no signal was detected in the nucleus or cytoplasm, that is, HMGB1 was secreted out of the cell. The number of cells in each of the four cases from three pictures of every group was counted to produce the quantification data.

Flow cytometry for CRT, ERp57, and PD-L1

The cells were treated with drugs as indicated for 4 h (CRT and ERp57 detection) or 24 h (PD-L1 detection) before harvesting. The cells were stained with anti-PD-L1 (124307, BioLegend), anti-CRT (1:1000, ab2907, Abcam), or anti-ERp57 (1:1000, ab10287, Abcam) and then with the Alexa Fluor 488-labeled secondary antibody (1:1000, 4412, CST). Isotype-matched immunoglobulin G (IgG) antibody (1:1000, ab171870, Abcam) was used as a control.

Analysis of HMGB1 and ATP release

Cells were treated with drugs as indicated for 24 h before the cell culture supernatants were collected. HMGB1 concentrations were assessed using a Mouse/Rat HMGB1 ELISA Kit (ARG81310, Arigo Biolaboratories). ATP secretion was detected using an Enhanced ATP Assay Kit (S0027, Beyotime).

T-cell depletion

For CD4 and CD8 T-cell depletion, anti-CD4 (clone GK1.5, BioXCell) and anti-CD8 (clone 53-6.7, BioXCell) antibodies were intraperitoneally injected at 500 μg per mouse on day 4 after tumor cell inoculation and then with follow-up injections of 250 μg per mouse on days 8, 12, and 16, respectively. The depletion efficacy was validated by flow cytometry detection of CD4+ or CD8+ T cells in peripheral blood mononuclear cells (PBMCs).

Immunohistochemistry

Tumor tissues were fixed in 4% paraformaldehyde (for staining HMGB1) or IHC Zinc Fixative (552658, BD Bioscience; for staining CD3, CD4, and CD8) at room temperature for 48 h before dehydration in an alcohol gradient and embedding with a Leica tissue embedder.

The slides were stained with primary antibody at 4 °C overnight as follows: anti-HMGB1 (1:100, 3935, CST), anti-CD3 (1:100, 4342633, Invitrogen), anti-CD4 (1:50, 100402, BioLegend), and anti-CD8 (1:25, 550281, BD Bioscience). The signals were detected using 3,3′-diaminobenzidine (DAB) (DAB-0031, MXB) and the cell nuclei were stained with hematoxylin.

Supplementary figures

Figure S1

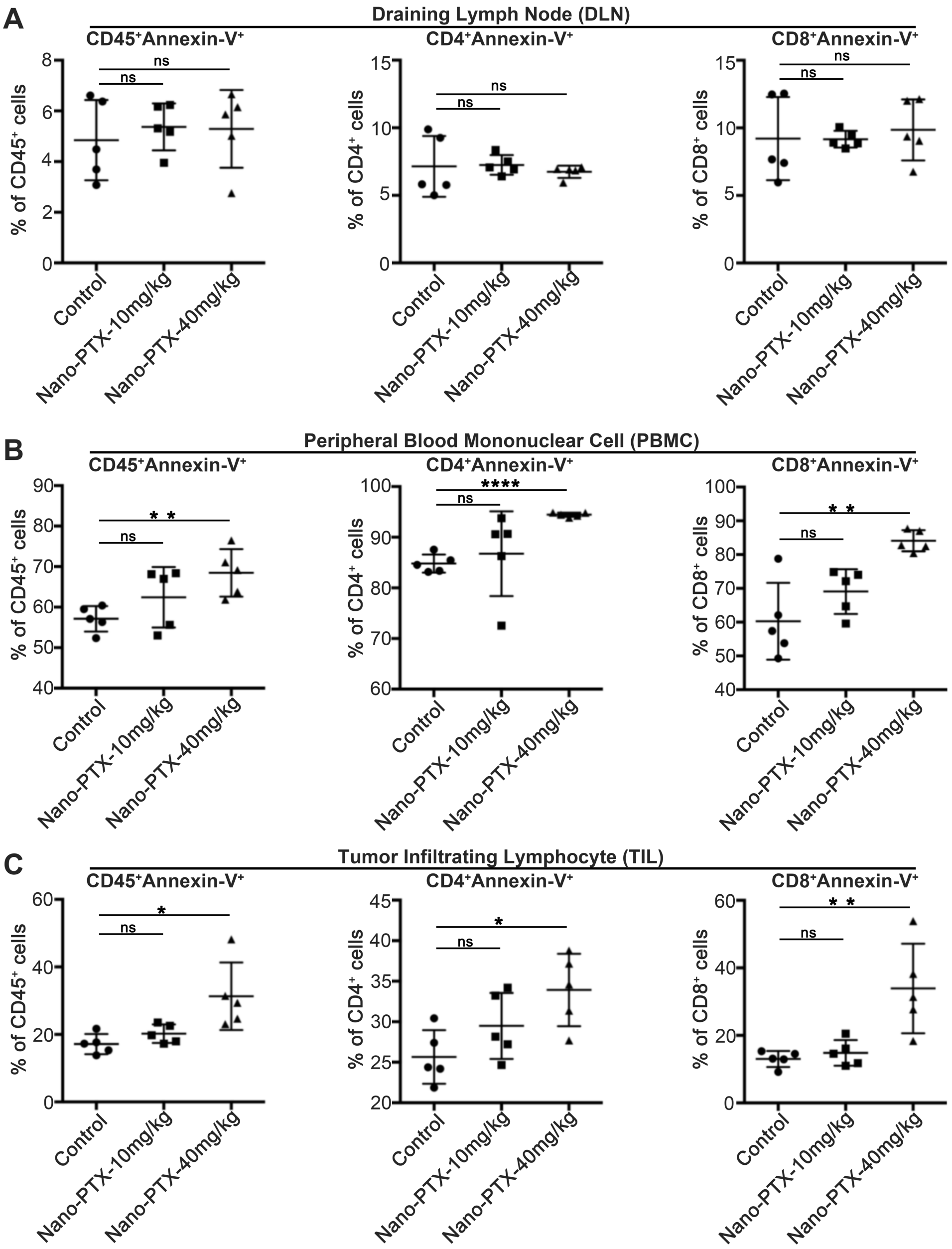

Supplementary Figure 1. The effects of different dose of nano-PTX on immune cells *in vivo*. Mice bearing CT26 were treated with nano-PTX (10 mg/kg) and nano-PTX (40 mg/kg). The percentage of apoptotic $CD45^+$ cells, apoptotic $CD4^+$, and apoptotic $CD8^+$ cells in draining lymph node (**A**), periphery blood (**B**), and tumor tissues (**C**) were shown. Mean ±SEM was shown. * $P < 0.05$, ** $P < 0.01$, **** $P < 0.0001$, ns (no statistical significance).

Figure S2

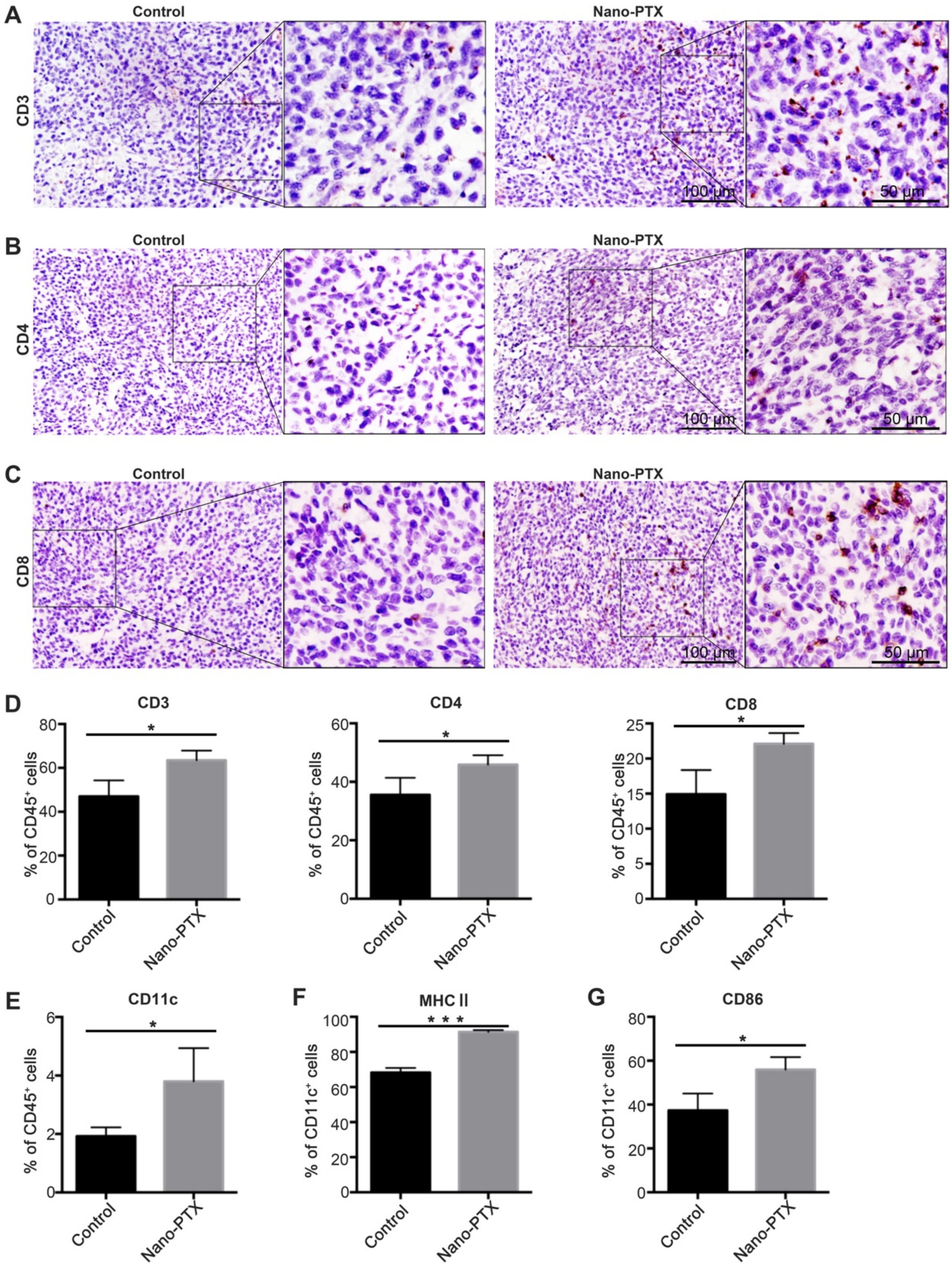

Supplementary Figure 2. Nano-PTX treatment changes immune cells infiltration in lymph node and tumor. Mice with established CT26 tumors were treated with nano-PTX as described in Figure 5G. **A-C** Immunohistochemistry staining of $CD3^+$ T cells (**A**), $CD4^+$ T cells (**B**), $CD8^+$ T cells (**C**) within CT26 tumor after nano-PTX injection (scale bar, 100 μm). An enlargement of local area was depicted (Scale bar, 50 μm). **D-G** Immune cells in lymph node were harvested and analyzed by flow cytometry on day 20 (n = 3 mice per group). The percentage of $CD3^+$, $CD4^+$, $CD8^+$ T cells (**D**) and DCs (**E**) in lymph node after nano-PTX treatment was shown. The activation status of DCs was measured by the expression of MHCII (**F**) and CD86 (**G**). Mean ±SEM was shown. * $P < 0.05$, *** $P < 0.001$.

Figure S3

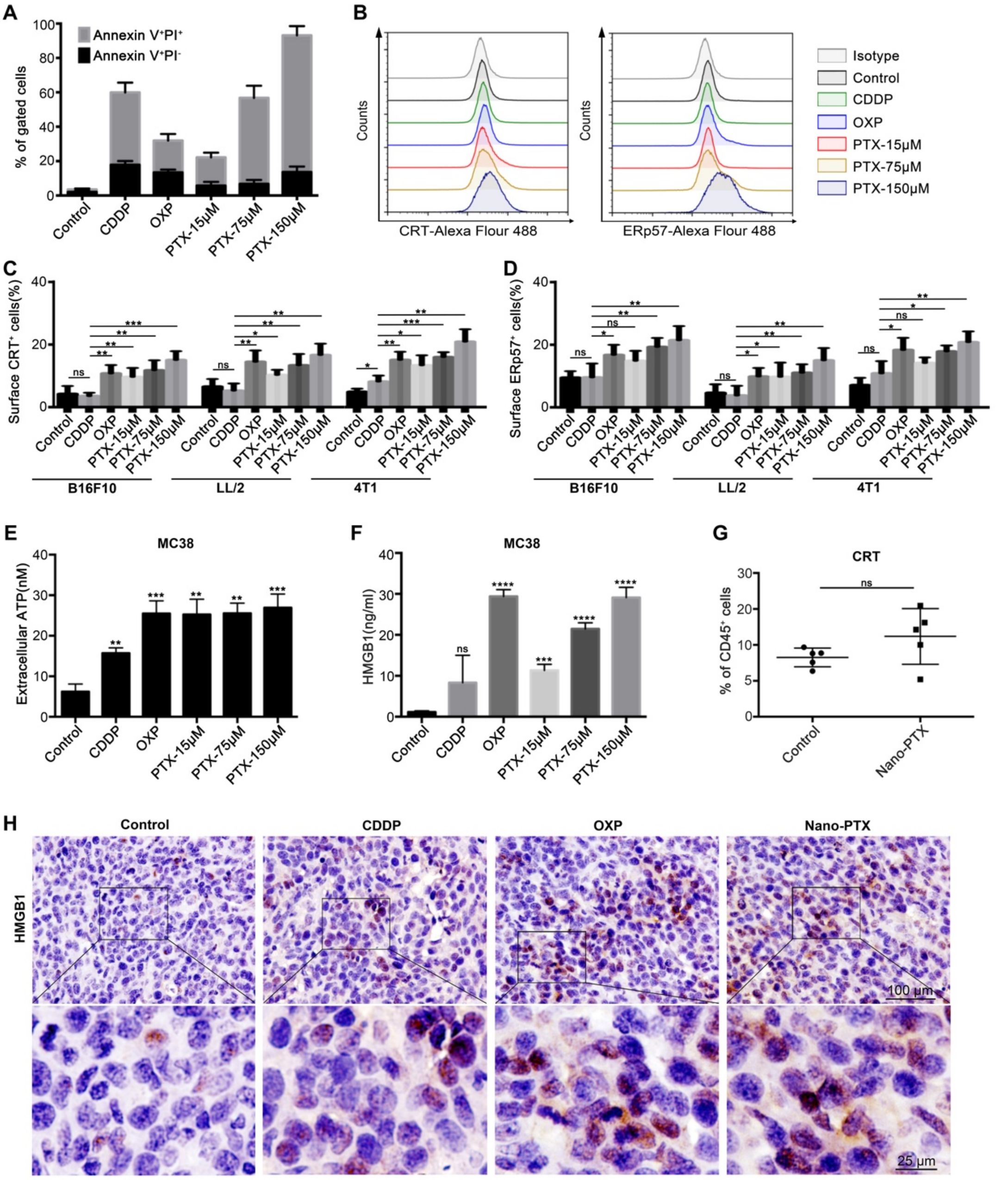

Supplementary Figure 3. PTX induces immunogenic cell death. A The apoptosis of CT26 cells treated with CDDP (150μM), OXP (300μM) or PTX, n = 3 replicates. **B** Representative histograms showed the expression of CRT and ERp57 on CT26 cells. **C-D** Flow-cytometry analysis of CRT (**C**) and ERp57 (**D**) on B16F10, LL/2 and 4T1 cells treated as indicated, n = 3 replicates. **E-F** The release of ATP (**E**) and HMGB1 (**F**) from MC38 cells treated with drugs for 24 h in the culture supernatants, n = 3 replicates. **G** Flow-cytometry detection of CRT on $CD45^+$ cells within CT26 tumor after nano-PTX injection, n = 5 mice per group. **H** Immunohistochemistry staining of HMGB1 within CT26 tumor after CDDP, OXP and nano-PTX injection (scale bar represents 100 μm). Mean ±SEM was shown. ** P < 0.01, *** P < 0.001, **** P < 0.0001, ns (no statistical significance).

Figure S4

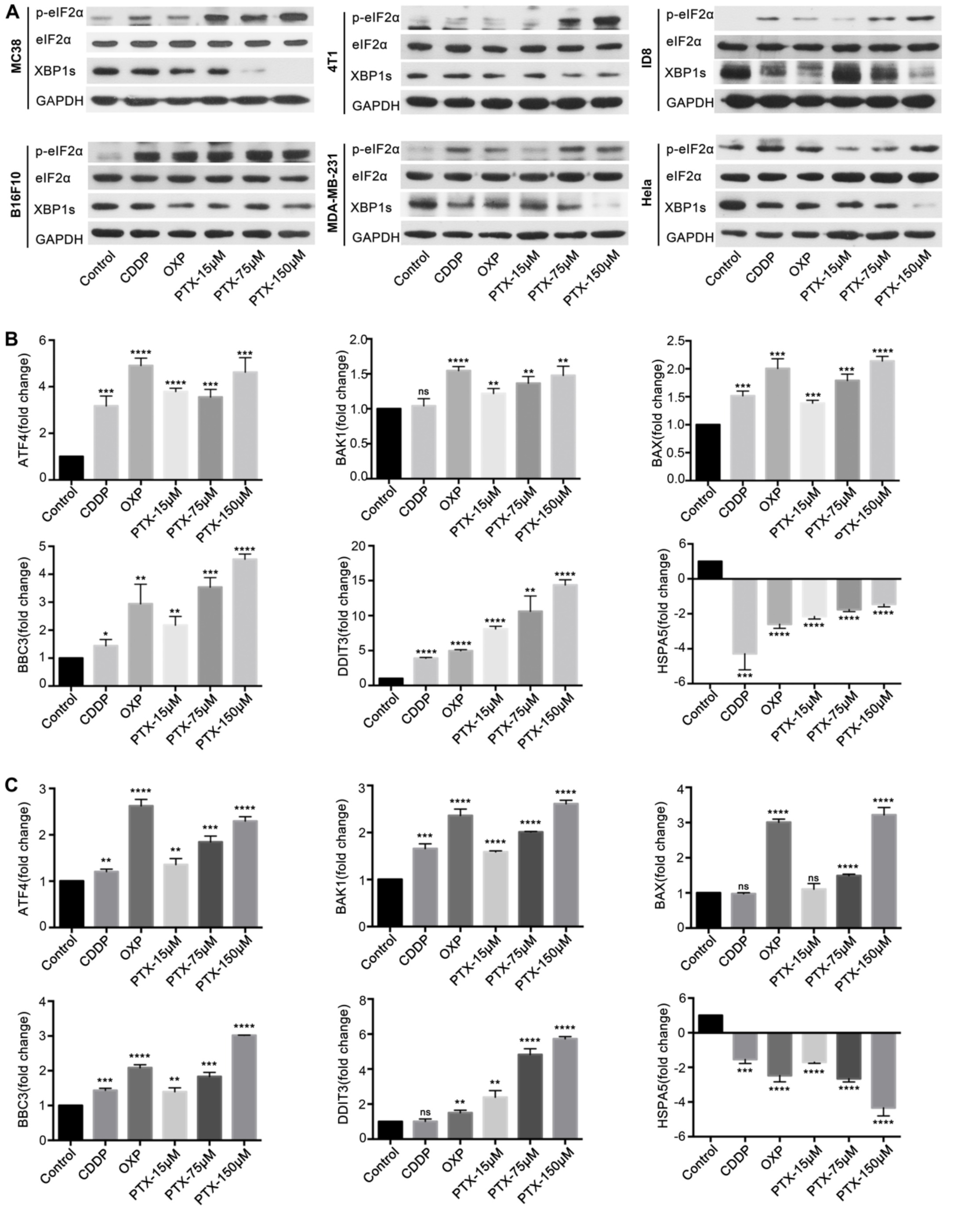

Supplementary Figure 4. PTX triggers ER stress response. A Western blot showing the protein level related to ER stress signaling pathway in MC38, 4T1, ID8, B16F10, MDA-MB-231 and Hela cells after treated with CDDP (150μM), OXP (300μM) and PTX for 4 h. **B-C**. RT-PCR analysis of ATF4, BAK1, BAX, BBC3, DDIT3 and HSPA5 expression level in CT26 (**B**) and MC38 (**C**) cells treated with indicated drugs for 4 h. n = 3 replicates. Mean ±SEM was shown. ** P < 0.01, *** P < 0.001, **** P < 0.0001.

Figure S5

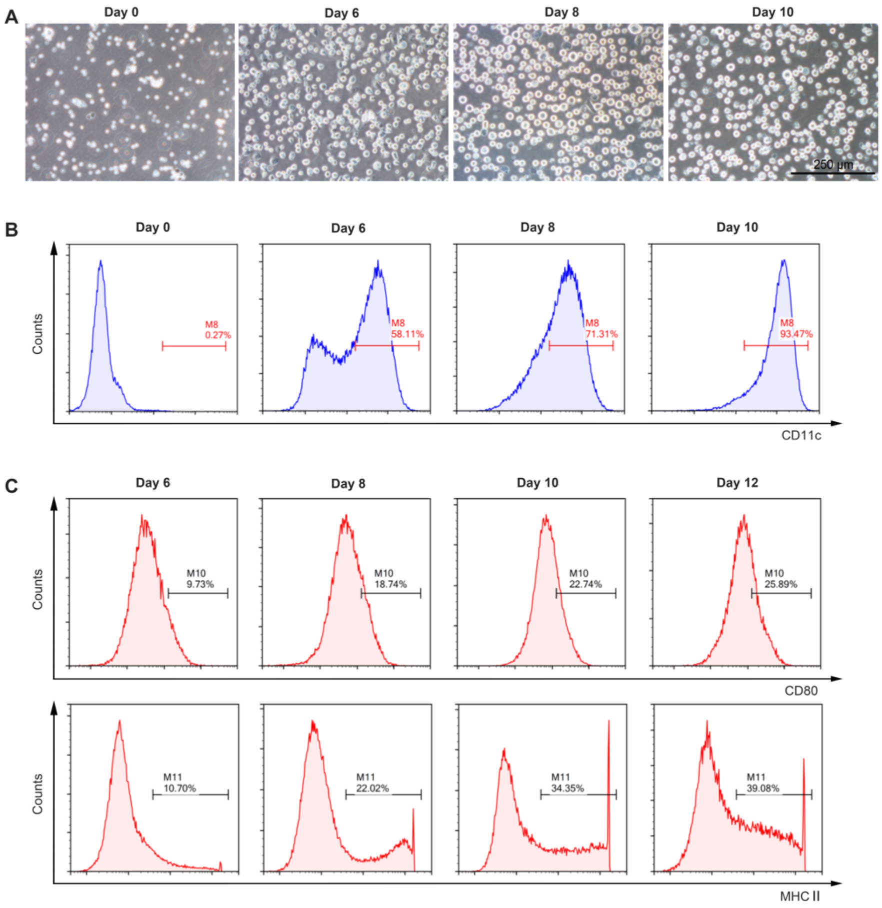

Supplementary Figure 5. Identification of bone marrow-derived DCs by cell morphology observation and flow cytometry. A Cell morphology of BMDCs, (Scale bar, 250 μm). **B-C** The expression of CD11c (**B**), CD86 and MHC Ⅱ (**C**).

Figure S6

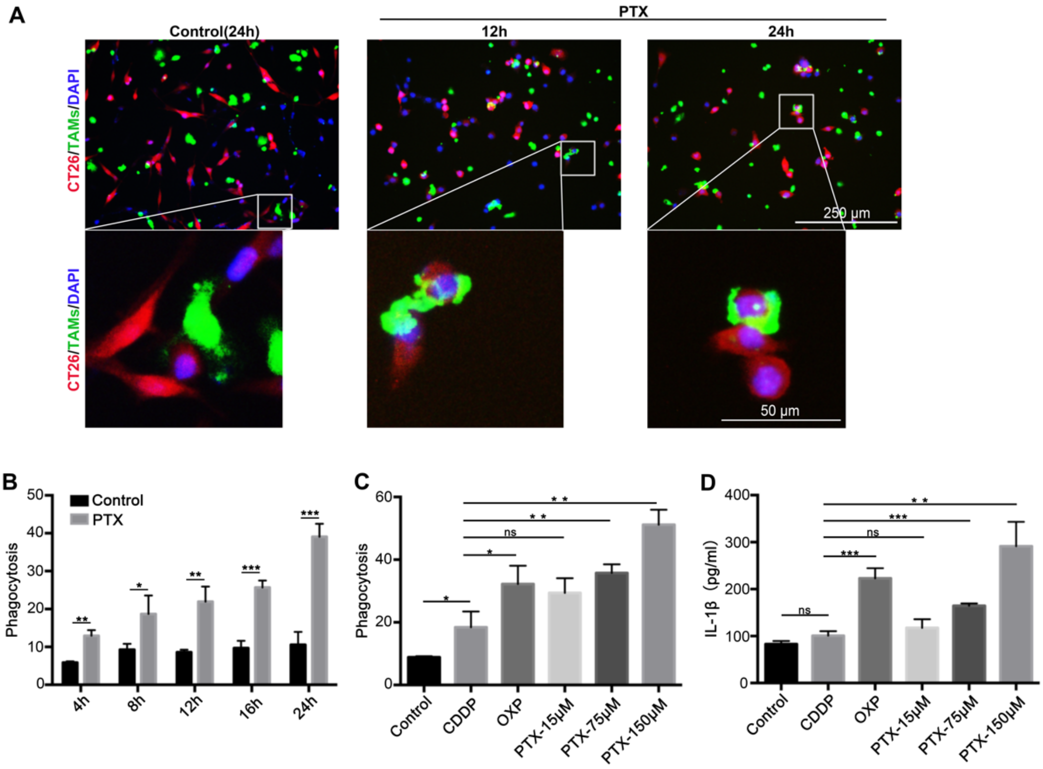

Supplementary Figure 6. PTX-treated tumor cells were readily phagocytosed by tumor-associated macrophages (TAMs). A Representative images showing TAMs (green) and PTX–treated CT26-RFP cells (red) at different time points of co-culture (Scale bar, 250 μm). An enlargement of the local area was depicted below (Scale bar, 50 μm). **B-C** The quantification of phagocytosis of CT26 (RFP) cell by TAMs (CFSE), n = 3 replicates. **D** The expression of IL-1β in the supernatant of co-culture CT26 cells with TAMs. Mean ±SEM was shown. * P < 0.05,** P < 0.01, *** P < 0.001, ns (no statistical significance).

Figure S7

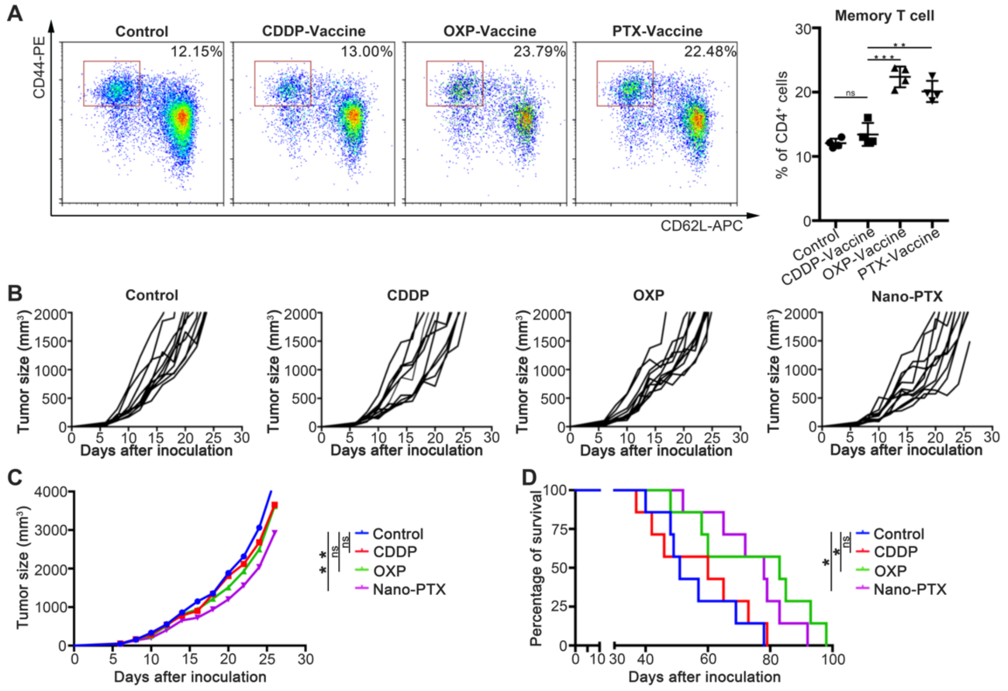

Supplementary Figure 7. PTX-prepared cell vaccines show therapeutic effects and nano-PTX treatment prolongs the survival of tumor-bearing mice. A The protective vaccination assay was conducted as described in Figure 5B and memory T cells ($CD4^{+}CD44^{high}CD62^{low/-}$) was detected by flow cytometry, representative flow images (left) and statistical graph (right) were shown. **B-C** The growth of CT26 subcutaneous tumor model treated with cell vaccines prepared with CDDP, OXP or nano-PTX, n=11 mice per group. **D** The overall survival of mice treated as Figure 5G was shown. Mean ±SEM was shown. * $P < 0.05$, **$P < 0.01$, *** $P < 0.001$, ns (no statistical significance).

Figure S8

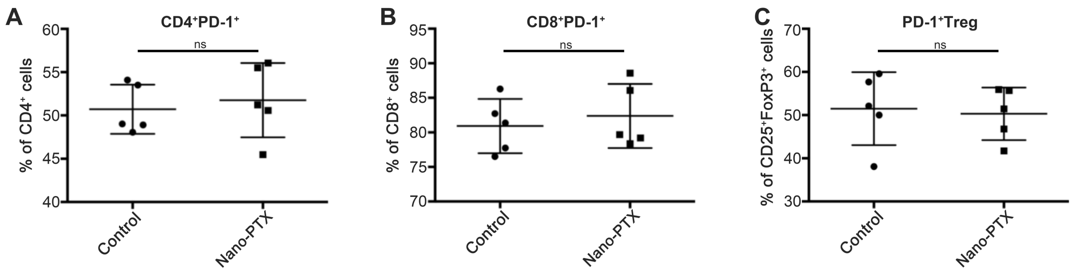

Supplementary Figure 8. The percentage of co-inhibitory molecule on immune cells in tumor. Mice with established CT26 tumors were treated with nano-PTX as described in Figure 5G, tumor cells were harvested and analyzed by flow cytometry on day 20. The PD-1 expression on $CD4^+$ cells (**A**), $CD8^+$ cells (**B**), and Treg cells (**C**) were showed, n = 5 mice per group. Mean ±SEM was shown, ns (no statistical significance).

Figure S9

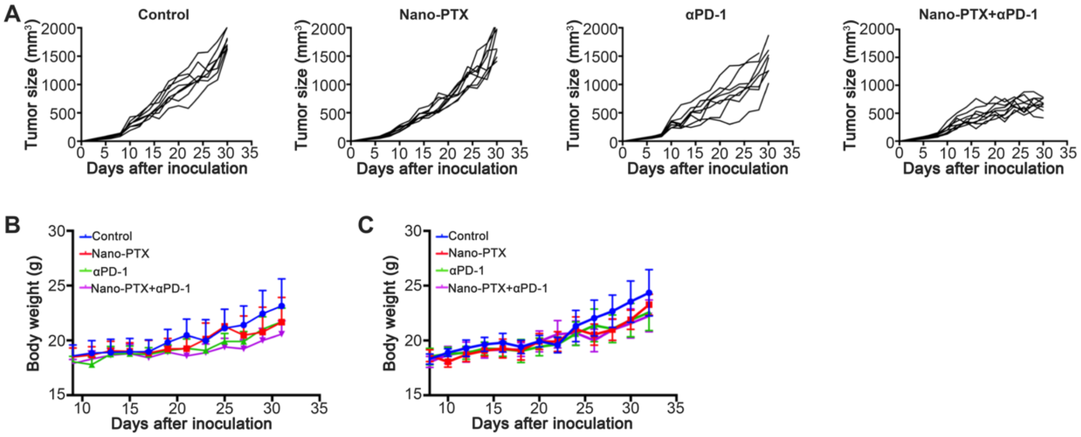

Supplementary Figure 9. Combination of nano-PTX and PD-1 antibody induces tumor regression. **A** Established 4T1 subcutaneous tumors were treated as indicated (Figure 7E-F). Individual tumor growth of mice was shown, n = 8~9 mice per group. **B** The body weight of MC38 tumor model in Fig7B. **C** The body weight of CT26 tumor model in Fig7G.

Figure S10

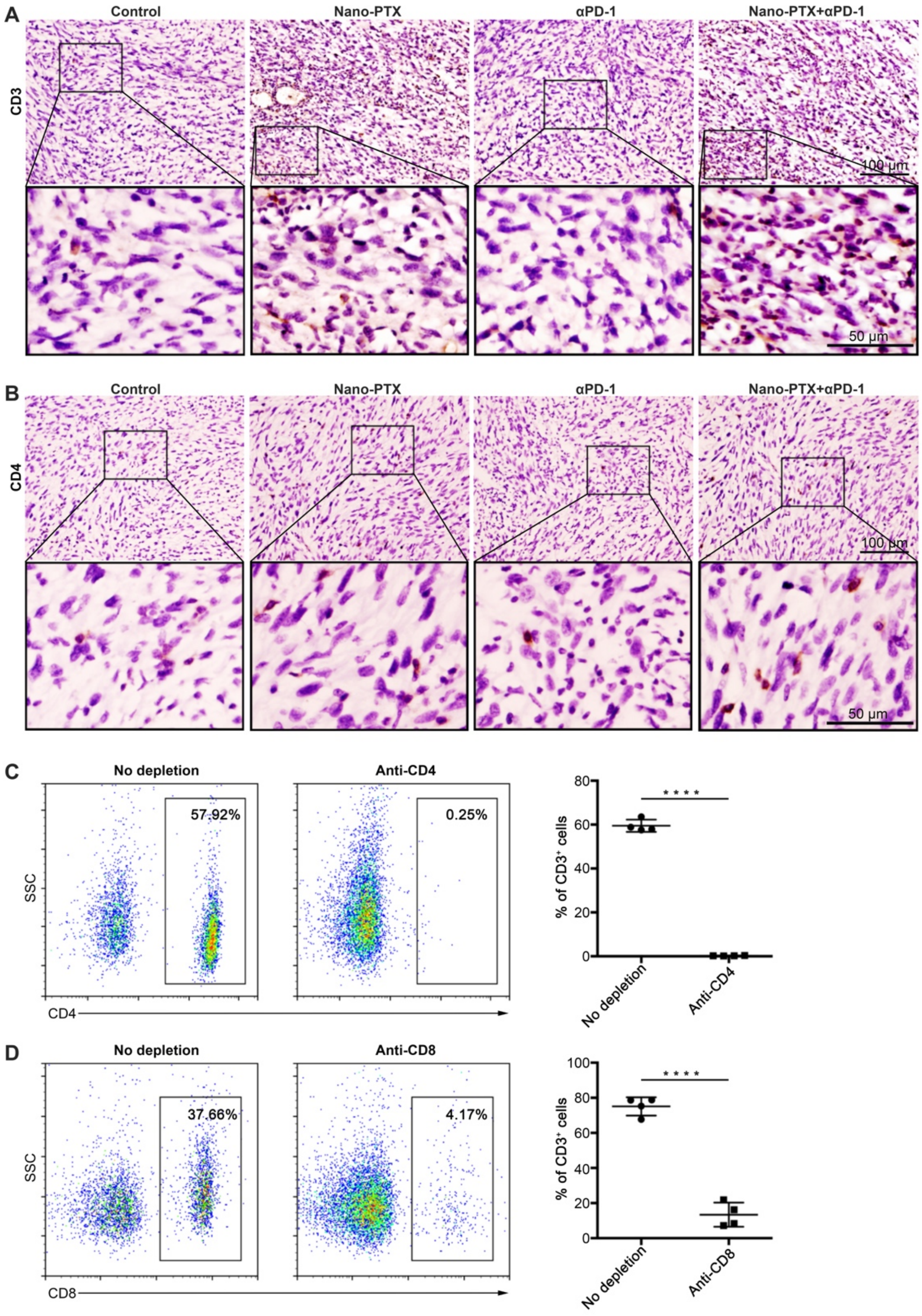

Supplementary Figure 10. The combination therapy increases the immune cells infiltration. A-B Mice with established MC38 tumors were treated with nano-PTX and PD-1 antibody as described in Figure 7A. Immunohistochemistry staining of CD3 (**A**) and CD4 (**B**) within CT26 tumor after treatment (scale bar, 100 μm). An enlargement of the local area was depicted below (Scale bar, 50 μm). **C-D** The percentage of $CD4^+$ T cell and $CD8^+$ T cell in PBMCs on day 18 after tumor cell inoculation, representative flow data and statistics were showed in (**C**) and (**D**) respectively, gated on $CD3^+$ cells, n = 4 mice per group. Mean ±SEM was shown. **** $P < 0.0001$.

Figure S11

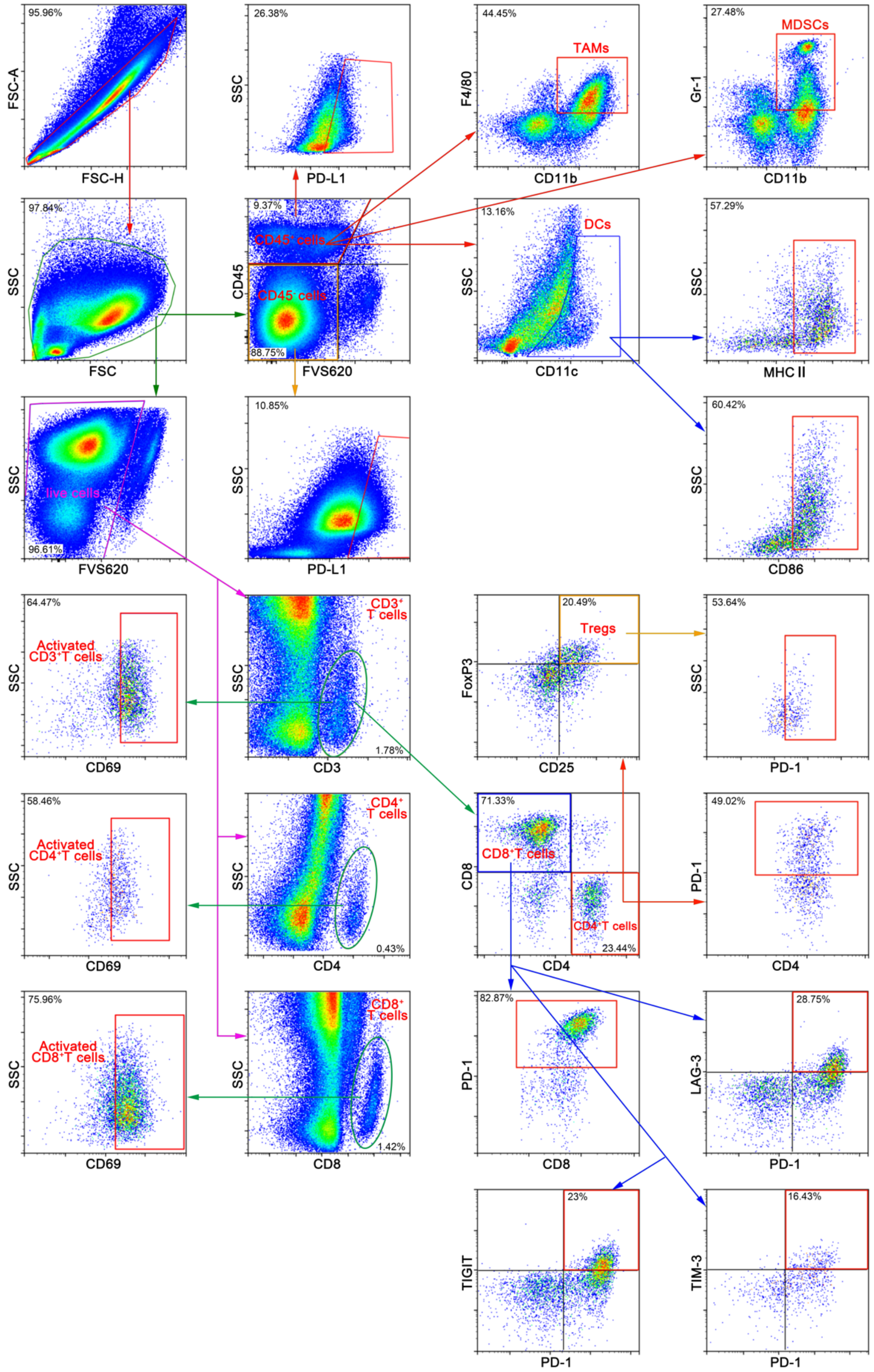

Supplementary Figure 11. Gating strategy for the analysis of immune cells and functional molecules in within tumor tissue. The gate was based on fluorescence minus one (FMO) control. Fixable Viability Stain 620 (FVS620) was used to discriminate live or dead cells.
